# Changes in Bacterioplankton Communities Resulting From Direct and Indirect Interactions With Trace Metal Gradients in an Urbanized Marine Coastal Area

**DOI:** 10.3389/fmicb.2019.00257

**Published:** 2019-02-22

**Authors:** Clément Coclet, Cédric Garnier, Gaël Durrieu, Dario Omanović, Sébastien D’Onofrio, Christophe Le Poupon, Jean-Ulrich Mullot, Jean-François Briand, Benjamin Misson

**Affiliations:** ^1^Mediterranean Institute of Oceanography (MIO), UM110, CNRS, IRD, Université de Toulon, Aix-Marseille Université, Marseille, France; ^2^MAPIEM, EA 4323, Université de Toulon, Toulon, France; ^3^Division for Marine and Environmental Research, Ruđer Bošković Institute, Zagreb, Croatia; ^4^LASEM-Toulon, Base Navale De Toulon, Toulon, France

**Keywords:** coastal ecosystem, metal contamination gradients, bacterioplankton community structure, functional prediction, co-occurrence network

## Abstract

Unraveling the relative importance of both environmental conditions and ecological processes regulating bacterioplankton communities is a central goal in microbial ecology. Marine coastal environments are among the most urbanized areas and as a consequence experience environmental pressures. The highly anthropized Toulon Bay (France) was considered as a model system to investigate shifts in bacterioplankton communities along natural and anthropogenic physicochemical gradients during a 1-month survey. In depth geochemical characterization mainly revealed strong and progressive Cd, Zn, Cu, and Pb contamination gradients between the entrance of the Bay and the north-western anthropized area. On the other hand, low-amplitude natural gradients were observed for other environmental variables. Using 16S rRNA gene sequencing, we observed strong spatial patterns in bacterioplankton taxonomic and predicted function structure along the chemical contamination gradient. Variation partitioning analysis demonstrated that multiple metallic contamination explained the largest part of the spatial biological variations observed, but DOC and salinity were also significant contributors. Network analysis revealed that biotic interactions were far more numerous than direct interactions between microbial groups and environmental variables. This suggests indirect effects of the environment, and especially trace metals, on the community through a few taxonomic groups. These spatial patterns were also partially found for predicted bacterioplankton functions, thus indicating a limited functional redundancy. All these results highlight both potential direct influences of trace metals contamination on coastal bacterioplankton and indirect forcing through biotic interactions and cascading.

## Introduction

Marine coastal areas are increasingly subjected to anthropogenic pressures that usually result in human-induced chemical contamination. Increasing demographic pressure and both terrestrial and marine activities threaten marine ecosystems (Halpern et al., 2008; The MerMex Group et al., 2011). Urban and industrial wastes (Levin et al., 2001; Oursel et al., 2013), biocides released by antifouling coatings (Turner, 2010) and fuel from nautical traffic (Callender, 2003) as well as historical pollution accumulated in the sediment compartment (Xu et al., 2014; Dang et al., 2015) are well-known and widespread examples. Such chemical contamination represents an increasing threat for marine coastal areas, and microorganisms inhabiting these ecosystems (Misson et al., 2016; Othman et al., 2017; Coclet et al., 2018).

Microbial communities have been shown to be a major component of marine planktonic ecosystems (Fuhrman and Azam, 1980), variable across space (Pommier et al., 2007; Ghiglione et al., 2012) and time (Fuhrman et al., 2006; Fortunato et al., 2012; Gilbert et al., 2012). Multiple environmental factors are known to drive temporal and spatial dynamics in bacterioplankton, such as salinity (Lozupone and Knight, 2007), temperature (Fuhrman et al., 2008; Gilbert et al., 2009), depth (Treusch et al., 2009), grazing, and predation (Zubkov and López-Urrutia, 2003) and resource availability (Fortunato et al., 2012; Gilbert et al., 2012; Chow et al., 2013).

Bacterioplankton communities have been largely characterized spatially or temporally in various environments, but rarely assessed over both spatial and temporal scales in a marine coastal area. These ecosystems are usually characterized by natural gradients linked to the continent-sea interface and if anthropized by a complex set of chemical contaminants, potentially affecting marine microbial communities. The links between benthic microbial communities and trace metal contamination have been extensively revealed (Gough and Stahl, 2011; Pringault et al., 2012; Quero et al., 2015; Misson et al., 2016). But how the marine bacterioplankton communities responds to trace metal exposure was scarcely investigated in coastal marine environments (Wang et al., 2015; Qian et al., 2017). Moreover, chemical perturbation on bacterioplankton communities is supposed to be complex because multiple trace metallic contaminations of the water column can result in additive, synergistic or antagonistic effects (Franklin et al., 2002; Lorenzo et al., 2002). Effects of trace metals can be dependant of hydrology, seasonal, and environmental conditions (Cloern, 2001). Furthermore, the impact of contamination on bacterioplankton may also be influenced by bacterio-phytoplankton coupling. Consequently, effects of contaminants on phytoplankton, which is sensitive to trace metal contamination (Coclet et al., 2018) might have indirect consequences for bacterioplankton.

Despite several studies describing microbial taxonomic diversity in marine coastal environments, functional diversity has not been investigated intensely (Jeanbille et al., 2016). Elucidating both taxonomic and functional diversity of microbial communities is the key to understand their roles in the ecosystem (Wang et al., 2016). Recent studies have related functional diversity of microbial communities to specific habitats (Dinsdale et al., 2008), water column zone (Louca et al., 2016), seasonal changes (Ward et al., 2017) and nutrient gradients (Thompson et al., 2017; LeBrun et al., 2018). However, relationships between trace metal contamination and functional profiles related to taxonomy in seawater ecosystems are still lacking.

Trace metals contamination was studied in a number of previous studies where authors mention benthic (Pringault et al., 2012; Besaury et al., 2014; Misson et al., 2016), phytoplanktonic (Othman et al., 2017) or freshwater communities (Wang et al., 2018). This study represents the first comprehensive assessment of bacterioplankton communities living in a contaminated marine coastal ecosystem. We combined in-depth physicochemical and geochemical characterizations of Toulon Bay seawater (north-western Mediterranean Sea), flow cytometry microbial enumerations and 16S rRNA gene-based high-throughput sequencing water samples over a weekly sampling campaign (weekly during 2015, June). The objectives of the study were (i) to study both spatial and temporal patterns in bacterioplankton communities along the contaminated Toulon Bay, (ii) to reveal the main environmental factors driving bacterioplankton communities, more precisely the contribution of trace metals through direct or indirect effects; and (iii) to identify the predicted functional profile’s response of bacterioplankton communities to chemical contamination in order to assess its influence on the ecosystem functioning.

## Materials and Methods

### Study Area and Sample Collection

Located in the south of France, Toulon Bay is divided into two basins by an artificial seawall: the Little Bay, which is semi-enclosed, and the Large bay, which opens onto to the Mediterranean Sea (Supplementary Figure S1). The Little Bay is characterized by a high level of anthropic activities (Navy harbor, ferry transport, industries, wastewater sewage, and aquaculture). Due to its location, the Little Bay is directly impacted by pollution and is less affected than the Large Bay by offshore hydrodynamics. This confers to the Little bay a higher trace metal contamination than the Large Bay as demonstrated previously in the sediments and with punctual measurements in the water column (Tessier et al., 2011; Coclet et al., 2018).

**FIGURE 1:**
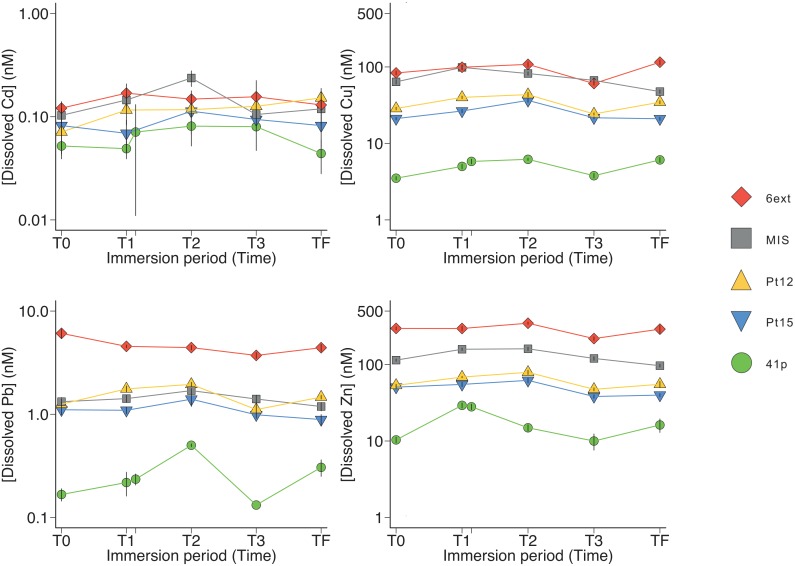
Dynamics of dissolved trace metals concentrations in surface seawater at the different sampling stations. Symbols and errors bars represent average and standard deviation, respectively.

Sampling was performed weekly from June 1st to 29th June, 2015. Two liters of seawater from the surface (1 m depth) and the bottom (1 m above sediment) of the water column were sampled at five sites across Toulon Bay (North-Western Mediterranean Sea, France), from the entrance of the bay to the north-western anthropized area (Supplementary Figure S1, Supplementary Table S1, and details in Supplementary Material). After the T1 sampling in site 41p, the French Navy performed an exercise that could have modified the water quality. In order to check that, we sampled again the water (T1b). The five sites were chosen to cover the whole range of dissolved metal contamination on the basis of trace metal distribution previously evaluated in Toulon Bay surface seawater (Coclet et al., 2018; Figure 1). Water samples were stored in a cooler and filtered through 0.2 μm polycarbonate membranes (Millipore). Filters were stored at −80°C until DNA extraction. Physico-chemical parameters including metals were weekly analyzed during the sampling period in order to characterize the environment (details in Supplementary Material). Standard oceanographic properties, including water temperature (°C), salinity, O_2_ (mg L^−1^ and %), pH, and chlorophyll *a* (chl*a*) (μg L^−1^) were measured on sites using a multi-parameter probe (Hydrolab DS5, OTT).

### Bacterial Community Abundance by Flow Cytometry

Subsamples of 10 mL of seawater were sampled at each site, depth, and date, filtered through a 90 μm nylon mesh, fixed with 0.25% (final concentration) glutaraldehyde on field and stored at −80°C until flow cytometry analysis. Autotrophic prokaryotes (*Synechococcus-*like), eukaryotic phytoplankton (picoeukaryotes and nanoeukaryotes) populations were characterized and enumerated using a BD Accuri^TM^ C6 (BD Biosciences) flow cytometer, as previously described (Coclet et al., 2018). Heterotrophic prokaryotes were enumerated after staining with SYBR Green as previously described (Cabrol et al., 2017).

### Bacterial Community Composition

DNA was extracted from the Millipore filters by a combination of enzymatic cell lysis (Ghiglione et al., 2009) and AllPrep DNA/RNA Mini Kit (QIAGEN) according to the manufacturer’s instructions. The protocol for the DNA extraction and the library preparation is fully described in Supplementary Material. We assessed bacterial community composition (BCC) by targeting the V4–V5 region of the 16S rRNA gene (Parada et al., 2016) and using Illumina Miseq 2 × 250 pb paired-end sequencing (Genoscreen, France).

Sequences were then demultiplexed and assigned to corresponding samples using CASAVA (Illumina). Forward and reverse reads were merged using PEAR 0.9.8 with default options (Zhang et al., 2014). Raw sequences were analyzed using MacQiime v.1.9.1 software (Caporaso et al., 2010). Briefly, barcode, primer, shorter sequences (<100 bp in length), and sequences with ambiguous base calls or homopolymer runs exceeding 10 bp were removed. The remaining sequences were assigned to operational taxonomic units (OTUs) and clustered at a 97% threshold using Uclust algorithm (Edgar, 2010), both closed and open reference OTU picking, based on the SILVA (release 128) database (Pruesse et al., 2007; Quast et al., 2013). Low abundance OTU (<0.005%) were filtered as recommended by Bokulich et al. (2013). Sequences classified as mitochondria or chloroplast were removed from the OTU table, corresponding to 3393 OTUs. A total of 349,009 reads were finally obtained representing 31,132 OTUs. OTU table was normalized by random subsampling to the smallest number of sequences (i.e., 4044). Samples with lower number of sequences were discarded (*n* = 16), 36 samples were remaining for following analyses (Supplementary Table S1). The 16S rRNA gene sequences have been deposited in the NCBI Sequence Read Archive (SRA) database under BioProject ID PRJNA514222^1^.

### Data Analysis

All plots and statistical analysis were done in R RStudio (R Core Team, 2015). Alpha diversity calculations and error estimates were averaged, using QIIME script *core_diversity_analyses.py* (Caporaso et al., 2010), including estimates of rarefaction, Chao1, equitability, Shannon, and Simpson’s diversity. In order to compare the sites based on their chemical composition, taxonomic and functional community profiles, seawater samples were sorted in a non-metric multidimensional scaling (NMDS). Pairwise dissimilarities were calculated using Bray–Curtis metrics. Each resulting dissimilarity matrix was used to visualize sample differences via NMDS ordination using the “vegan” package in R (Oksanen et al., 2016). To test the null hypothesis that there were no significant difference between the groups discriminated according to sampling stations, sampling dates, and depth, similarities were analyzed by global and pairwise PERMANOVA tests, using “vegan” and “RVAideMemoire” packages, respectively.

To identify taxa that discriminated the sampling sites, the linear discriminant analysis (LDA) effect size method (LEfSe) (Segata et al., 2011) was used. This was performed with the LEfSe online tool in the Galaxy framework, using all default setting for data formatting and LDA effect size (Goecks et al., 2010). A similarity percentage (SIMPER) was run with a 90% cutoff and used to rank the percent contribution of individual biomarker to the dissimilarity between site differences (Clarke, 1993).

To investigate the relationships between BCC and measured environmental variables, Spearman rank correlation analysis, and redundancy analysis (RDA) were performed using the “vegan” package in R (Oksanen et al., 2016) (details in Supplementary Material). Briefly, for variable reduction and in order to create an efficient model from the most significant explanatory variables, vegan’s ordistep function were applied and among the 31 explanatory variables, 20 significant variables (*P* < 0.05) were kept for the following analyses The RDA model was tested by performing partial RDA, and variation partitioning to test the significance of the contribution of both groups of variables and each individual variable.

Interactions among the OTUs and between these OTUs and the 31 environmental variables were evaluated using CoNeT (Co-occurrence Network) plugin 1.1.1.beta (Faust et al., 2012), a plugin in a Cytoscape software 3.4.0. A similarity matrix was built with different metrics (Spearman correlation and Kullback–Leibler distance and a mutual information score) from OTUs. This initial network was re-defined by randomization. A permutation matrix representing a null distribution was obtained by resampling OTUs as described in Faust et al. (2012). In a permutation step, edge specific *p*-values were computed; however, for the final network, *p*-values of an edge were merged into one *p*-value according to Brown’s method. In the final step, the Benjamini–Hochberg multiple testing correction was applied (*P* < 0.05). Network characterization was evaluated using betweenness centrality (BC) and closeness centrality (CC) (details in Supplementary Material). Highly connected clusters were identified using the MCODE plugin version 1.4.0.beta2 (Smoot et al., 2011). Network characterization were evaluated using different topological indexes generated by Network Analyzer plugin.

Functional profiles were predicted from obtained 16S rRNA gene data using Tax4Fun (Aßhauer et al., 2015) based on KEGG category.

## Results

### General Characteristics of Toulon Bay Seawater Samples

An increase in the trace metal concentrations was rationally observed from a reference site open on the sea with low metal concentrations (41p) to enclosed sites in the most anthropized area (MIS and 6ext) with high level of contaminations (Figure 1). The highest trace metal concentrations found in surface seawater were Zn, Cu, Pb, and Cd, while other metals/metalloids (As, Ba, Be, Cs, Cr, Mn, Sb, Sn, Ti, U, and V) were kept to concentration levels close to the geochemical background (Figure 1 and Supplementary Table S2). At the surface of seawater, Cd, Cu, Zn, and Pb concentrations were 6, 33, 35, and 48 times higher in 6ext water than in 41p water, respectively. These enrichment factors were quite similar (8, 25, 43, and 73 times) at the bottom of the seawater (Supplementary Figure S2). For both surface and bottom seawater, the concentrations of trace metals/metalloids were relatively stable during the sampling period (Figure 1, Supplementary Figure S2, and Supplementary Table S2). The most contaminated sites MIS and 6ext were significantly discriminated from the reference site 41p (PERMANOVA, *P* < 0.01) (Supplementary Figure S3A) and from sampling date (Supplementary Figure S3B) but not with depth (Supplementary Figure S3C).

Seawater temperature increased gradually during the studied period at all five sites, from 18.8°C–21.2°C in T0 to 22.0°C–24.3°C in TF (Supplementary Figure S4). Additionally, temperature was lower (1.9 ± 0.04°C) in bottom than in surface seawater. Surface salinity levels demonstrated low variations (38.4 ± 0.11). A very punctual drop was recorded in all sites at T2, down to 36.7 in MIS (Supplementary Figure S4). Chlorophyll *a* in surface seawater were lower in 41p (0.16 to 0.31 mg L^−1^) than in other sites (0.59 to 2.08 mg L^−1^). Additionally, chlorophyll *a* concentrations peaked in all sites at the middle of the survey. Except for 41p, chlorophyll *a* concentrations were higher at the bottom seawater than at the surface (Supplementary Figure S4). On average, in surface seawater, higher concentrations of DOC were found in 6ext (1.4 ± 0.07 mg L^−1^) compared to 41p (1.2 ± 0.06 mg L^−1^) (Supplementary Figure S4). Similarly, higher concentrations of TN were found in MIS (0.15 ± 0.02 mg L^−1^) compared to 41p. (0.08 ± 0.02 mg L^−1^) (Supplementary Figure S4). No significant difference was found in dissolved oxygen concentrations (mg L^−1^, %), and pH between both all five sites and all sampling date (Supplementary Table S2). NO^3-^ and PO_4_^3-^ concentrations of were below the detection limit (0.02 and 0.020 μM, respectively) in a majority of samples. As a consequence, they are not presented and will not be considered further.

### Bacterial Community Dynamics

Heterotrophic bacterioplankton abundances estimated using flow cytometry are shown in Supplementary Figure S5. We observed an increase in the bacterioplankton abundance from the reference site 41p (3.8 × 10^5^ to 4.2 × 10^5^ cell mL^−1^) to the most enclosed site 6ext (8.0 × 10^5^ to 1.4 × 10^6^ cell mL^−1^) in both surface and bottom seawater. Additionally, heterotrophic bacterioplankton abundance was stable in 41p during the experiment whatever the depth, while strong dynamic was observed in both surface and bottom 6ext seawater, with highest abundances in T2 and T3, respectively.

Over the sampling, observed richness, and Chao1 index (Supplementary Figure S6) were significantly higher in the uncontaminated site 41p and declined to less than half compared to 6ext (ANOVA, *P <* 0.01). In contrast, Shannon, Simpson, and evenness indexes did not significantly vary between sampling sites (Supplementary Figure S6).

**FIGURE 2:**
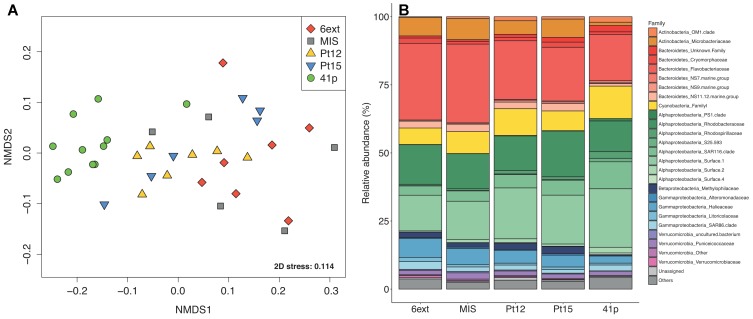
Spatial variations of bacterioplankton community structure. **(A)** Non-metric dimensional scaling (nMDS) ordination based on Bray–Curtis dissimilarity for 16S rRNA gene libraries between the different sampling sites. Each symbol corresponds to a distinct site, time, and depth point. **(B)** Bar plot showing the average family-level contribution for each site. Families that on average comprised less than 1% of the libraries are grouped as “Others.” Colors indicate different classes within phyla.

NMDS results showed a significant segregation of bacterioplankton communities between both sampling sites (PERMANOVA; *P* = 0.001) and dates (PERMANOVA; *P* = 0.024) but not with depths (PERMANOVA; *P* = 0.983) (Figure 2A and Supplementary Table S3). More precisely, pairwise PERMANOVA tests (Supplementary Table S3) confirmed significant differences between the reference 41p site and all other sites (PERMANOVA; *P* < 0.05) and significant differences between 6ext and Pt12–Pt15 (PERMANOVA; *P* < 0.05).

The most abundant phyla ( > 1% of all sequences across all samples) were *Proteobacteria* (53 ± 1.1%), followed by *Bacteroidetes* (28 ± 1.1%), *Cyanobacteria* (9.1 ± 0.7), *Actinobacteria* (6.1 ± 0.6), and *Verrucomicrobia* (3.0 ± 0.3%). At the family level (Figure 2B), *Flavobacteriacea* comprised the majority (22 ± 1.1%) of the total sequences, followed by *Alphaproteobacteria* groups SAR11 Surface 1 clade (18 ± 1.1%), *Rhodobacteraceae* (13 ± 0.65%) and SAR116 clade (6.1 ± 0.49%). *Cyanobacteria*, *Actinobacteria*, and *Verrucomicrobia* phyla were dominated by *Synechococcus* Family I (9.1 ± 0.72%), *Microbacteriaceae* (4.9 ± 0.71%), and *Puniceicoccaceae* (1.7 ± 0.19%). Archaea represented less than 0.1% of the total sequences, thus they were considered as “Others” in this study and variations of this group have not been taken into consideration.

**FIGURE 3:**
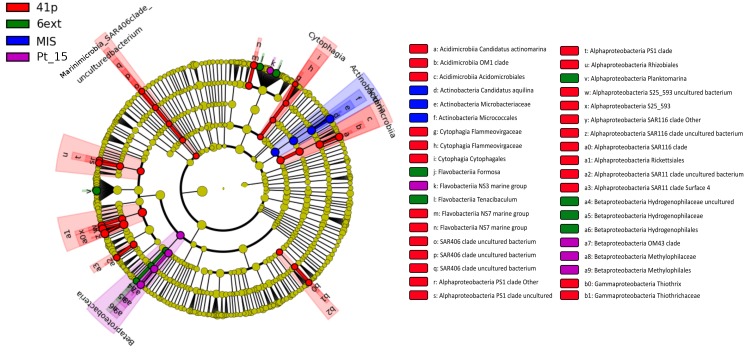
Taxonomic tree generated using LefSe analysis (*P* < 0.05, LDA effect size > 2) highlighting the biomarkers that statistically differentiate sampling sites. No biomarker was evidenced for the sampling site Pt12. The roots of cladogram stand for the domain (i.e., Bacteria and Archaea), and concentric circles represent the following taxonomic levels until the tips standing for genera.

Clear shifts in the relative abundance of individual bacterioplankton OTUs were observed between sampling sites. Using LEfSe analysis, we determined 43 differentially abundant taxa between four sites. 41p and 6ext, the two most geochemically contrasted sites, exhibited the highest number of taxonomic indicators (25 and 6, respectively) (Figure 3). For 41p site, the most significantly enriched bacterioplankton sequences were Acidimicrobiia *Candidatus Actinomarina*, members of the family SAR406, 7 Alphaproteobacteria OTUs and Gammaproteobacteria *Thiothrix*. Actinobacteria *Candidatus Aquilina* and both Flavobacteriia *Formosa* and Betaproteobacteria *Hydrogenophilaceae* were found to be overrepresented in MIS and 6ext, respectively. SIMPER analysis revealed that, on average, 13 biomarkers were identified as major contributors to differences between sites detected in the NMDS. The higher number of biomarkers identified as major contributors was found between 41p and the other sampling sites (between 17 and 32 biomarkers) (Supplementary Table S4). Overall, each biomarker contributed to at least 1% of the dissimilarity between sites. *Candidatus Aquilina*, PS1 clade, NS7 marine group, *Planktomarina* and *Tenacibaculum*, notably, drove together more to 32% of disparity between cluster 41p and the other clusters (Pt15, 6ext, and MIS).

### Bacterial Community Composition (BCC) in Relation With Environmental Variables

**FIGURE 4:**
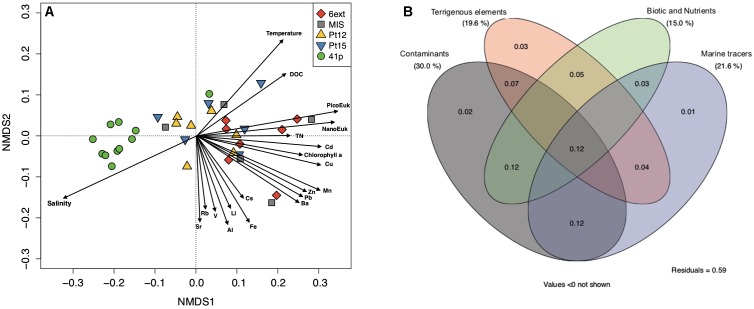
Contributions of environmental variables to spatial differentiation in bacterioplankton community structure. **(A)** Redundancy analysis (RDA) ordination diagram of the first two axes for bacterioplankton community composition. The percentage of the spatial variation in community structure explained by each axis is indicated in parentheses after the axis label. The constrained sets of environmental variables analyzed are indicated as vectors. NanoEuk: Nanoeukaryote abundance; PicoEuk: Picoeukaryotes abundance. **(B)** Venn diagram showing the variation partitioning (%) of the spatial variations in bacterioplankton community structure among four environmental datasets (Contaminants, Terrigenous elements, Marine tracers, and Nutrient and Biotic).

Among the 31 environmental variables available for multivariate analysis, 20 parameters were selected by pairwise correlations. The first and the second axes of RDA captured 47.1% and 6.4% of BCC variance, respectively (Figure 4A).

Variation partitioning provided more details about the relative contributions of nutrients, contaminants, terrigenous, and “others” factors in the observed spatial changes in BCC (Figure 4B). The combination of the four sets of explanatory variables explained 41% of BCC variation across sites. Among the set of explanatory variables, contaminants were the main contributors to variations in BCC (30%). Contaminants also appeared to have significant influence on BCC through their interaction with the other sets of variables. The contribution of terrigenous (19.6%), nutrient and biotic (15.0%) and “marine tracers” (21.6%) variables were also significant but to a lower extent than the contribution of contaminants variables. The highest individual contributions were attributed to Mn (1.2%), DOC (1.2%), salinity (1.1%), Zn (1.1%), and Cd (1.0%) (Supplementary Table S5). Thus, among the five explanatory variables which contributed the most to the biological variation, three variables were contaminants (Mn, Cu, and Cd).

**FIGURE 5:**
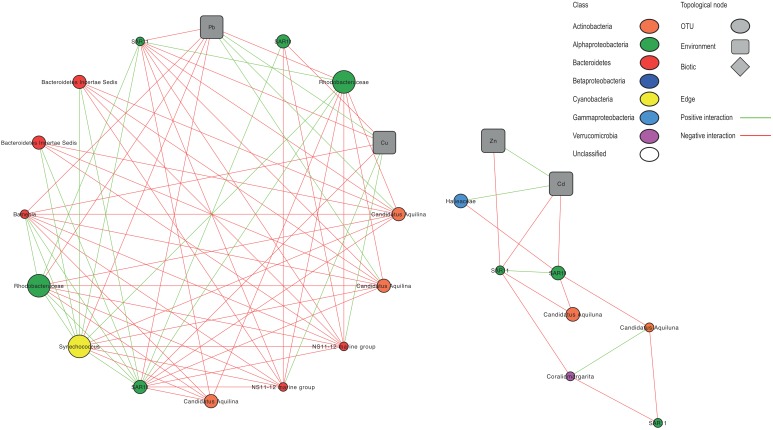
Co-occurrence subnetworks of bacterial taxa and environmental variables derived from the OTU table and showing only statistically significant correlations. Node size correspond to the relative abundance of the OTU. Nodes colors correspond to main bacterioplankton classes. Green and red lines represent positive and negative correlations, respectively.

Network analysis resulted in a global network representing 158 OTUs and 21 environmental variables significantly connected. Among 1664 edges, 92% were inter taxa edges, and 8% represented taxon-environment interactions. Most of the connections (67%) corresponded to negative interactions. To visualize the results, eight subnetworks were extracted from the global network (Figure 5 and Supplementary Figure S7).

Globally, by plotting BC vs. CC, 12 OTUs with the highest values for both parameters (arbitrarily determined as BC > 0.02 and CC > 0.5) were selected, representing putative keystone species within Toulon Bay (Supplementary Table S6). Both of these indices indicate that SAR11 Surface 1 clade and *Rhodobacteraceae* (Alphaproteobacteria), *Synechococcus* (Cyanobacteria), *Candidatus Aquiluna* (Actinobacteria), and *Balneola* (Bacteroidetes) were central in this network. Most of Alphaproteobacteria, including SAR11, SAR116, and *Rhodobacteraceae*, and Cyanobacteria *Synechococcus* were positively correlated with each other, but negatively correlated with Bacteroidetes (NS11-12 and NS5 marine group, *Cryomorphaceae* and *Balneola*), and Actinobacteria (*Candidatus Aquilina*).

In the subnetworks, there were 19 correlations with contaminants (8 with Pb, 6 with Cu, 3 with Cd, and 2 with Zn) and two correlations with chlorophyll *a*. Several taxa that were identified as being key drivers of community dissimilarity using LEfSe analysis were highly correlated to contaminants. In particular, the Alphaproteobacteria *Rhodobacteraceae* and SAR11, the Bacteroidetes *Balneola* and the Cyanobacteria *Synechococcus* showed negative correlations with at least one of the contaminants (Cd, Cu, Pb, or Zn). Conversely, Actinobacteria *Candidatus aquilina* and Bacteroidetes NS11-12 marine group were positively correlated with Pb and Cu, respectively. Gammaproteobacteria *Haliaceae* was positively correlated with Cd.

### Bacterioplankton Predicted Functional Capabilities of Microbial Communities

**FIGURE 6:**
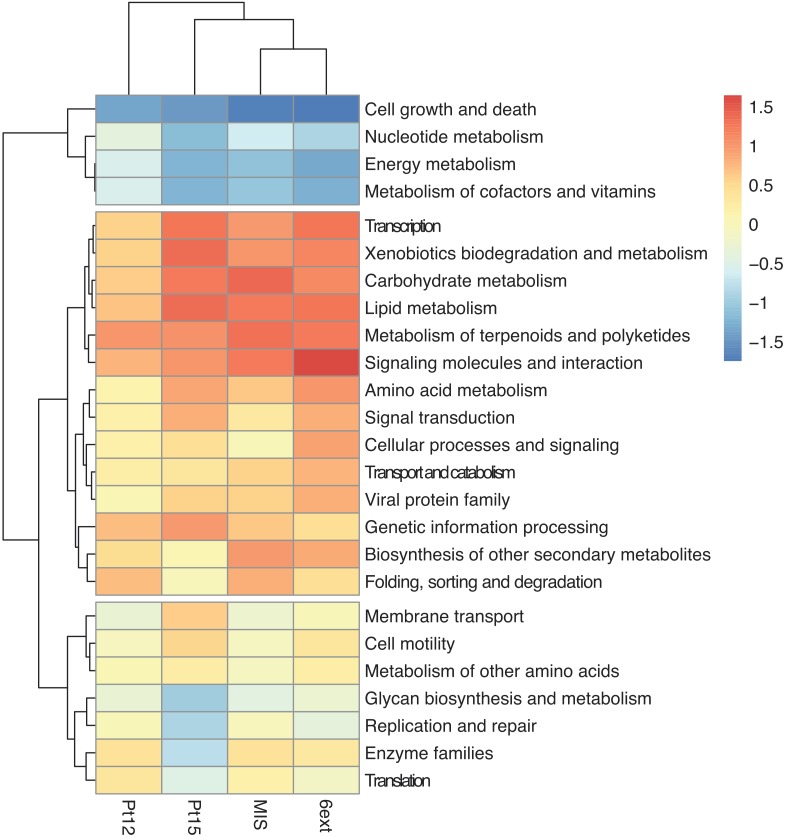
Heatmap based on the main KEGG pathways predicted at the different sampling sites. Frequencies of predicted KEGG categories were normalized with reference to the uncontaminated site 41p. Enrichments correspond to the positive values and red colors, depletions correspond to negative values and blue colors.

The functional profiles of bacterioplankton communities was predicted using Tax4Fun (Aßhauer et al., 2015). Predicted functions were classified as KEGG orthologs (KOs) resulting in the identification of 3619 KOs across all samples, using on average, 60 ± 12% of total OTUs (Supplementary Table S7). Clustering showed that the differentiation of the sampling sites based on microbial predicted function profiles followed the chemical gradient described earlier, from the reference site toward the most enclosed and contaminated 6ext site (Figure 6). Compared to the uncontaminated site (41p), used here as a reference, some KEGG pathways seemed overrepresented in contaminated sites: Carbohydrate metabolism, Xenobiotics biodegradation and metabolism, Metabolism of terpenoids, and polyketides. Other KEGG pathways were found to be underrepresented as “Cell growth and death,” “Nucleotide metabolism,” “Energy metabolism,” and “Metabolism of cofactors and vitamins.”

## Discussion

Bacterioplankton community composition and associated functions in highly metal contaminated ecosystems were scarcely studied (Nayar et al., 2004; Boyd et al., 2005; Pringault et al., 2016), even less using NGS techniques (Wang et al., 2015; Qian et al., 2017). This study was performed in a multi contaminated coastal area, Toulon Bay, which presents far wider contamination gradients than natural environmental gradients. By combining seawater physicochemical and geochemical analyses with patterns of bacterioplankton diversity and predicted functions, we provide an innovative insight into bacterioplankton ecology allowing to better understand how trace metals could shape communities.

### Trace Metal Contaminations as Drivers of Bacterioplankton Communities

Marine coastal areas are seen as valuable sentinel ecosystems, providing signals that reflect anthropogenic chemical pollution (The MerMex Group et al., 2011). Concentrations of trace metals in the Toulon Bay were found to be at least comparable or higher to those reported in aquatic ecosystems receiving high contamination loads (Boyd et al., 2005; Pringault et al., 2012). The high enrichment factors (6- to 234-fold) compared to the geochemical background of the Mediterranean Sea (Morley et al., 1997) indicated significant anthropogenic inputs that could be attributed to numerous recent (e.g., large boat traffic, harbor activities, antifouling coatings) (Turner, 2010) and historical events (2nd World War) (Tessier et al., 2011). Thus, we hypothesized a potential effect of trace metals on the bacterioplankton communities.

As previously reported in coastal waters (Nayar et al., 2004; Caroppo et al., 2006; Pringault et al., 2016), bacterioplankton was more abundant in the most contaminated zones (MIS and 6ext) than in the uncontaminated site 41p. Bacteria have been reported to have a wide range of metal detoxification or resistance mechanisms by sequestering, excluding or precipitating metals (Nies, 1999). The higher abundance in contaminated sites could therefore suggest that the community is well adapted. Moreover, bioavailability of trace metals could also contribute to explain the absence of bacterioplankton abundance decrease in the most contaminated sites in term of abundance. Trace metals bioavailability is strongly controlled by the presence of dissolved organic ligand, especially in marine waters (Louis et al., 2009; Bruland et al., 2013). We considered here DOC concentrations and the dissolved fraction of trace metals, which is closer to the bioavailable fraction than the total concentration (Paquin et al., 2002; Cindrić et al., 2017) and consequently allow a more reliable assessment of their roles. Nevertheless, we neither evaluated the speciation of the main trace metals nor DOC composition in this study. More work will be thus needed to state about the respective contributions of bioavailability modulation and community adaptation in the bacterial abundance increase in Toulon Bay.

While bacterioplankton diversity did not show any significant trend along the contamination gradient, the community richness (Chao1 index) was shown to decrease progressively from the uncontaminated site (41p) toward the most contaminated sites (MIS and 6ext). A similar pattern was observed in South-Eastern Australia in metal- and PAH-contaminated sediments (Sun et al., 2012) as well as in contaminated sediments in the Northern Adriatic Sea (Korlević et al., 2015). This may be interpreted in light of classis disturbance theory that predict that in highly disturbed ecosystems, richness would decrease and a few species would dominate (Odum, 1985; Allison and Martiny, 2008). As a matter of fact, sensitive species are expected to be lost whereas tolerant species benefit from emptying ecological niches (Atlas et al., 2015). Thus, the multi-contamination appeared to have disruptive effect on the alpha diversity of the bacterioplankton communities.

Gillan et al. (2005) demonstrated that alpha-diversity alone is a poor indicator of ecosystem stress in chronically polluted systems, as the proliferation of new tolerant species can lead to a recovery of the diversity. As in many coastal sites, microbial assemblages in our study were dominated by a few families *Flavobacteriaceae*, SAR11 Surface 1 clade, *Rhodobacteraceae*, SAR116 clade, *Synechococcus* Family I, and *Microbacteriaceae*. This is a core of generalist families in marine seawater and its members have been found abundant in numerous other marine coastal studies (Chow et al., 2013; Cram et al., 2015). Despite overall similarities, BCC was strongly affect by trace metal contamination, the five sampling sites clearly exhibited distinct microbial community’s structures, along low-to-high contamination continuum. This observation is also in agreement with the disturbance hypothesis, as discussed for alpha diversity, and tends to complement previous observations of the structural role of contaminants for bacterioplankton in other coastal areas (Pringault et al., 2016; Marisol et al., 2018; Wang et al., 2018).

The uncontaminated site (41p) was characterized by higher relative abundances of OM1 clade (Acidimicrobiia), SAR11, Rickettsiales, Rhizobiales, and Rhodobacteraceae (Alphaproteobacteria), Thiothrichaceae (Gammaproteobacteria), and *Synechococcus* (Cyanobacteria). Correspondingly, a number of previous works reported that *Synechococcus* were sensitive to high trace metal levels (Cassier-Chauvat and Chauvat, 2014; Coclet et al., 2018). Additionally, SAR11, Rickettsiales, Rhizobiales, and *Synechococcus* are well known to be characteristic of marine zones away from anthropogenic influence (Gilbert et al., 2009, 2012; Fuhrman et al., 2015).

The most contaminated sites (MIS and 6ext) were enriched in *Candidatus Aquiluna* (Actinobacteria), *Formosa* and *Tenacibaculum (*Flavobacteriia) as well as one from Sphingobacteriales NS11-12 marine group (Bacteroidetes). Networks analysis showed also that *Planktomarina* (Alphaproteobacteria), *Hydrogenophilaceae* (Betaproteobacteria) and Halieaceae (Gammaproteobacteria) were positively associated with contaminants, in agreement with the distribution of these groups along the contamination gradient. Previous studies in freshwater or marine sediment reported that *Hydrogenophilaceae* and *Candidatus Aquiluna* (Microbacteriaceae) were positively correlated to high trace contaminant levels (Acosta-González et al., 2013; Garris et al., 2018). The increase of Actinobacteria and Betaproteobacteria in the most contaminated site was not unexpected, as they are widely recognized to play a key role in oil degradation (Acosta-González et al., 2013) and heavy metals transformation, especially, in the case of nutrient enrichments (Garris et al., 2018). Microbacteriaceae (Actinobacteria) were widely reported as members of highly copper-polluted sediment (Besaury et al., 2014) or Zn-polluted freshwater (Ni et al., 2016). Albarracín et al. (2010) have shown that Actinobacteria members isolated from copper-contaminated sediment are able to significantly diminish the bioavailable copper throughout bioaccumulation. Members of the Bacteroidetes members, especially *Flavobacteria* families are also known to represent one of the most abundant groups of bacteria in coastal areas, especially in contaminated sites due to their ability to tolerate toxic effects of certain metals (Sun et al., 2013: Korlević et al., 2015). The Bacteroidetes NS11-12 marine group have been detected mainly in marine habitats (Meziti et al., 2015), unfortunately without clear ecological implications. At a finer scale, for most of clusters, members of both Alphaproteobacteria (mainly SAR11) and Bacteroidetes (mainly *Flavobacteriia*) groups describe above, exhibit the highest number of edges with high level of connections within each group in the network analysis. These important findings display that Alphaproteobacteria would highly influence the functioning of low contaminated site while Bacteroidetes would highly influence the functioning of contaminated zones.

Most of studies on bacterioplankton communities have focused on community diversity and composition responses to perturbation (Jeffries et al., 2016). The effect of chemical contaminations on bacterial community functionalities have only been investigated in freshwater ecosystems (Wang et al., 2018; Sjöstedt et al., 2018). In a highly polluted area such as Toulon Bay, the functional stability of microbial communities could be challenged. Major predicted bacterial functions including those necessary to basic metabolisms were conserved across the chemical gradient. Functional recovery occurred because of species replacement or change in relative abundance of taxa, i.e., functionally redundant rare species could become abundant in response to the contaminations. This is in agreement with the replacement scenario (Comte and Del Giorgio, 2011), and previous findings showing that the rare biosphere is an important reservoir for recruitment of bacterial taxa during environmental change (Sjöstedt et al., 2012; Comte et al., 2014). However, both a decrease in some critical functions (cell growth and death or energy metabolism) and an increase in functions associated to multiple stress resistance (e.g., xenobiotic biodegradation and metabolism, signaling molecules and interactions, secondary metabolites metabolism) remarkably exhibited that bacterial communities in the most polluted sites developed specific functions to face their environment. As the sites are not physically separated, there was no discontinuity in both communities and functions but a clear gradient of the relative components throughout the metal gradient. Metal contaminated environments were shown to exert a high selective pressure toward the transfer of several genes, developing thus resistance against high metal concentrations (Azarbad et al., 2016). Only two field studies on long-term metal polluted areas have shown shifts in microbial community structure and functions or increased occurrence of resistance genes that have made microbial communities resistant to toxic metals concentrations (Hemme et al., 2010; Kang et al., 2013). However, both taxonomic affiliation of metal-specific metabolic traits and the currently available KEGG database are insufficient to validate the hypothesis that heavy metal stress is a key environmental factor shaping the function of microbial communities.

### Implication Regarding Others Local Selective Pressures

Although trace metal gradient seemed to be the main driver of the bacterioplankton community features, other local selective pressures could explain a significant proportion of their variance. Indeed, bacterioplankton communities in coastal areas greatly vary in space because of sharp gradients in salinity, nutrients, depth, among other properties (Fuhrman et al., 2006; Gilbert et al., 2009; Fortunato et al., 2012).

A very similar trend could have been observed if DOM quantity or quality varied and became more labile in the most contaminated sites. Indeed, an increase in DOM lability often leads to the development of a copiotrophic response of the bacterial community, selecting a limited number of bacterial taxa, and temporarily reducing microbial diversity (Nelson and Wear, 2014; Pedler et al., 2014). The variation partitioning performed in our study is in agreement with this hypothesis, since DOC appeared as one of the most influent drivers of community structure variations. Thus, organic matter spatio-temporal dynamics could indeed be an important driver of microbial communities in Toulon Bay. However, according to the literature, only a part of the bacterial taxa significantly enriched in the most contaminated sites of Toulon Bay is known as copiotroph. Indeed, while OM60 (NOR5) clade (Halieaceae), *Candidatus Aquiluna* (Microbacteriaceae), and *Planktomarina* (Rhodobacterales) are known to benefit from labile DOM (Yan et al., 2009; Spring et al., 2015), Alphaproteobacteria members (SAR11 and AEGEAN-169 marine group) and Bacteroidetes (*Balneola* and *Aquibacter*) are rather known as oligotrophic groups (Cram et al., 2015; Sipler et al., 2017). Copiotrophs are expected to outcompete oligotrophs, so the fact that presence of groups identified as copiotrophs suggest that each has capitalized on reduced competition following trace metal contamination. Taken together, all these arguments point out the major and predominant influence of the multi-contamination gradient on microbial diversity and represent a significant demonstration of the potential influence of human activities on marine microbial life. Since we only considered inorganic contaminants, we cannot rule out the potential influence of organic contaminants such as PAH, PCBS, or organometals which were already measured in the sediment of Toulon Bay (Misson et al., 2016).

Organic contaminants as polyaromatic hydrocarbons could explained more precisely the distribution of OTUs (Qian et al., 2017). This hypothesis could be confirmed by the strong enrichment in xenobiotics biodegradation and metabolism, carbohydrate metabolism and metabolism of terpenoids and polyketides in the highest contaminated sites. Naphthalene degradation, benzoate, and aminobenzoate biodegradation were found to be higher in the most contaminated zones and is consistent with the finding of previous studies (Wang et al., 2018).

Finally, another striking result is that the multi-contamination gradient in the water column of Toulon Bay represented a significant perturbation, affecting microbial diversity all along our survey. This observation differs from what was observed previously in surface sediment of Toulon Bay (Misson et al., 2016), suggesting that the contemporary contamination in the water affects more strongly microbial communities that the large historical contamination trapped in the sediment. Such difference could be linked to the probably higher dispersal rate in the water column than in the sediment, i.e., 3.4 days for a total renewal of the water of Toulon Bay (Dufresne et al., 2014). Thus, this higher dispersal rate could tend to reduce the adaptive abilities of bacterioplankton, leading to a higher sensitivity to contamination when compared to the longer-term stability of the sediment.

### Biotic Interactions as Evidences of Indirect Effects of Trace Metals

The network indices (BC and CC) and large numbers of nodes (179) and edges (1664) between taxa in our network analysis suggest that SAR11, Rhodobacteraceae, Synechococcus, and Microbacteriaceae play a key role in Toulon Bay seawater. Despite some connections between OTUs and trace metals, there were far more numerous connections among OTUs. This suggests that trace metals significantly influence the dynamics of only a few microbial groups, and could rather influence indirectly, via biotic interactions, the whole community in Toulon Bay seawater. Community structure is known to be influenced by interaction among species (Lima-Mendez et al., 2015). Some of these interactions could be cooperative interspecific interactions (Eiler et al., 2012; Chow et al., 2013) or competitive interactions (i.e., competition, niche partitioning, grazing, or parasitism) (Fuhrman et al., 2008; Berdjeb et al., 2018) in a multispecies community.

Keystone OTUs play important roles in the regulation of network interactions (Wright et al., 2012) and their loss may increase a community’s vulnerability to perturbation. Thus, the strong decline of Alphaproteobacterial and Cyanobacterial keystone OTUs, especially, along the trace metal contamination gradient could have indirect effect on the BCC by modifying biotic interactions and favor opportunist or tolerant taxa (Lawes et al., 2016). This is in agreement with community turnover and the development of copiotrophic groups highlighted above.

Additionally, in network analysis, positive correlation between on the one hand pico- and nanophytoplankton abundances, as well as chlorophyll *a*, and on the other hand trace metals concentrations describe potential conditions that may favor specific groups of bacterioplankton. Indeed, Fuhrman et al. (2015) explained that weekly timescale is appropriate for studying bacterial dynamics associated with phytoplankton, which have a direct influence on the composition of bacterial communities via cross-feeding interactions or the effect of toxins, as well as indirect influence (e.g., oxygen depletion). Phytoplankton succession may influence the dynamics of the bacterioplankton community, as differences in the phytoplankton structure lead to differences in quantity and quality of exuded organic matter that can be used by the bacterial community for growth (Marisol et al., 2018). Consequently, effects of pollutants on phytoplankton or bacterioplankton might have indirect consequences for the counterpart, depending on the possible interactions between both compartments.

In summary, Toulon Bay offered a remarkable study area allowing to address trace metal multi-contamination impacts on prokaryotic community shaping throughout contrasted but connected sites. Where trace metal contamination increased, bacterioplankton abundance was higher together with a reduction of only community richness. Seawater contamination induced significant bacterioplankton communities shifts, with Alphaproteobacteria (SAR11, Rickettsiales, Rhizobiales, and Rhodobacteraceae) and *Synechococcus* dominance where trace metal concentrations were the lowest, whereas Flavobacteria, Betaproteobacteria (Hydrogenophilaceae) and Gammaproteobacteria (Halieaceae), especially including copiotrophic organisms, dominated in highly contaminated sites. In addition, this study showed that human-induced disturbances can have an effect on predicted functional potential of marine coastal bacterioplankton, and that the response can differ according to the type of functions. However, observed functional redundancy lead to think that the effect of trace metals could be complex, highly context-dependent and depending on biotic interactions. While our results provide a better understanding of anthropogenic influence on coastal ecosystems and the response of microbial communities, future studies are needed to elucidate the contribution of DOC, phytoplankton-bacteria specific interactions and organic pollutants on bacterioplankton communities in Toulon Bay.

## Originality-Significance Statement

Human-induced chemical contamination gradients challenge our understanding of microbial communities’ ecology, especially in the very dynamic marine coastal environment. Through the spatio-temporal investigation of microbial diversity dynamics in a model ecosystem, this work highlights contributions of anthropogenic trace metal contamination to marine bacterioplankton ecology. Identify the key aspects of originality and significance that place the work within the top 10% of current research in environmental microbiology.

## Author Contributions

CG, J-FB, and BM proposed and designed the study. CC, CG, GD, CLP, J-UM, J-FB, and BM organized and performed field sampling. CC, CG, GD, DO, SD, CLP, and BM analyzed the samples. CC, CG, J-FB, and BM interpreted the results. All authors participated in manuscript redaction, thus approving the publication of the content.

## Conflict of Interest Statement

The authors declare that the research was conducted in the absence of any commercial or financial relationships that could be construed as a potential conflict of interest.

## Footnotes

1 https://www.ncbi.nlm.nih.gov/biosample/10721482
