# Supplementary material for: Changes in Bacterioplankton Communities Resulting From Direct and Indirect Interactions With Trace Metal Gradients in an Urbanized Marine Coastal Area
Source: Front Microbiol. 2019 Feb 22;10:257. doi: 10.3389/fmicb.2019.00257 (PMC6395402; doi:10.3389/fmicb.2019.00257)
Supplement: Supplementary file 1 [file Data_Sheet_1.pdf]

Supplementary Information

Changes in bacterioplankton communities resulting from direct and indirect interactions with trace metal gradients in an urbanized marine coastal area

Clément Coclet[1,2], Cédric Garnier[1], Gaël Durrieu[1], Dario Omanović[3], Sébastien D'onofrio[1], Christophe Le Poupon[1], Mullot Jean-Ulrich[4], Jean-François Briand[2], Benjamin Misson[1,*]

[1] *Université de Toulon, Aix Marseille Université, CNRS, IRD, Mediterranean Institute of Oceanography (MIO), UM110, France.*

[2] *Université de Toulon, MAPIEM, EA 4323, Toulon, France.*

[3] *Ruđer Bošković Institute, Division for Marine and Environmental Research, Bijenička 54, Zagreb, Croatia.*

[4] *LASEM-Toulon, Base Navale De Toulon, BP 61, 83800 Toulon, France.*

[*]: corresponding author; email: misson@univ-tln.fr; tel: +33 494 146 746; Université de Toulon, CS60584, 83041 Toulon Cedex 9

1. **Supplementary Material**

Environmental parameters.

A description of the pre-cleaning protocol, the storage and the measurement of nutrients (dissolved organic carbon: DOC (mgC.L^{-1}), total nitrogen: TN, and nitrate: NO_3^- (mgN.L^{-1})) and dissolved trace metal (Cd, Co, Cu, Ni, Pb and Zn) (nM) concentrations is detailed in Coclet et al. (2017). Additionally, Trace metal concentrations in seawater samples were analysed also by HR ICP-MS (Element 2, Thermo Finnigan; at IRB, Zagreb). Measurements were performed after dilution of the samples in a 2 % HNO3 solution (dilution factor 10) spiked with a stock solution of indium as an internal standard (final concentration: 10 µg/L). Standards of elements were prepared in the matrix matching media (UWSV, 2 % HNO3) for the assessment of element concentration by means of external calibration. Phosphate concentrations (PO_4^{3-}, mgP.L^{-1}) were determined using a UV 1800 Shimadzu Spectrophotometer and micro cell Quartz SUPRASIL®, 50-mm light path (Hellma Analytics), based on the Rimmelin and Moutin (2005) MAGnesium Induced Coprecipitation (MAGIC) method.

DNA extraction, PCR amplification and sequencing preparation. Nucleic acids were extracted by a combination of mechanic and enzymatic cell lysis techniques applied directly to polycarbonate membrane filters using lysis solution (50 nM glucose, 10 nM EDTA, 25 nM Tris pH 8.0), followed by consecutive incubations with lysozyme (20 mg.mL^{-1}, 37°C, 45 min) and SDS 10% with proteinase K (20 mg.mL^{-1}, 55°C, 60 min). DNA was purified using AllPrep DNA/RNA Mini Kit (QIAGEN) according to the manufacturer's instructions (Ghiglione et al., 2009). DNA concentration was measured using a NanoDrop ND-100.

Microbial communities were characterized using a dual index sequencing approach with primers targeting the V4 region of the bacterial and archaeal 16S rRNA genes. The primer construct consisted of a six-base barcode and the 16S forward primer 515Y (GTGYCAGCMGCCGCGGTAA) and the 16S reverse 926R (CCGYCAATTYMTTTRAGTTT) (Parada et al., 2016). PCR reactions were carried out in a 25 µL reaction mixture composed of DNA sample, GoTaq® Long PCR Master Mix (PROMEGA, France), and 0.1 µM of each primer. PCR reactions were thermo-cycled using the following protocol: 95 °C for 2 min; and 30 cycles at 95 °C for 30 s, 56 °C for 30 s and 72 ° C for 90 s; with a final extension at 72 °C for 10 min. PCR products were then purified with AMPure XP beads (Agencourt Bioscience) and quantified by Quant-iT™ PicoGreen™ dsDNA Reagent (Life Technologies) as described by the manufacturer's instructions. Purified products from samples were pooled in equimolar concentrations. In total, 52 libraries were paired-end (2 × 250bp) sequenced on the MiSeq (Illumina) at Genoscreen (Lille, France).

Redundancy analysis. To investigate the overall effects of abiotic parameters on BCC, we performed Spearman rank correlation analysis and redundancy analysis (RDA) of the relative taxon abundances as well as of the relative functional group abundances, against 31 explanatory variables: temperature, salinity, chlorophyll *a*, DOC, TN (Fig S1), dissolved oxygen ($mg.L^{-1}$ and %), pH, eukaryotic phytoplankton (pico- and nanoeukaryotes) abundances (Table S3), dissolved Cd, Cu, Pb and Zn (Fig S2), and the 17 chemical elements from Table S2. Prior to the RDA, the "ordiR2step" function in Vegan (Oksanen et al., 2012) was used to automatically select constraints in the model using forward stepwise selection and Akaike's information criterion with 999 permutation tests at each step. We repeated the forward stepwise selection with the bacterial community from each sampling site. Among the 31 explanatory variables, 20 significant variables ($P<0.05$) were kept for the following

analyses. To evaluate the significance of the redundancy axes, we used an ANOVA test for RDA with Monte Carlo permutation test (999 permutations). Variation partitioning was used to evaluate whether pure trace metal variables affected the BCC independently of the effect of other variables (Oksanen et al., 2012). In Dang et al. (2015), the geochemical analysis of Toulon Bay revealed that three-main groups were clearly identified: (i) marine elements (with Sr, notably), (ii) contaminants and (iii) terrigenous elements. Based on this study, all significant explanatory variables were divided into four groups. Thus, firstly, we separated the 'pure contaminants' (A) effect as referring to the trace metals, Ba, Cd, Cu, Pb, Zn, Mn. Secondly, we separated "terrigenous elements" (B) (Li, Rb, Cs, Al and Fe) and "nutrient and biotic variables" (C) (DOC, TN, chlorophyll *a*, picoeukaryote and nanoeukaryote densities). Thirdly, we generated a set of variables related to "other variables" as 'marine tracers" (D) (V, Sr, Temperature and Salinity). Each group of explanatory variables was tested independently as well as in combination. To verify the robustness of our results for various taxonomic resolutions, we considered the taxonomic composition at the OTU, genus, family, order, class and phylum level.

2. Supplementary Figures

Fig. S1 Map of the studied site Toulon Bay with the location of the sampling sites. Solid and dashed lines represent a seawall and treated sewage submarine outlets, respectively.

Fig. S2 Dynamics of dissolved trace metals concentrations in bottom seawater at the different sampling stations. Symbols and errors bars represent average and standard error (n = 3), respectively.

Fig. S3 Nonmetric dimensional scaling (nMDS) ordination based on Bray-Curtis dissimilarity for explanatory variables (temperature, salinity, chlorophyll a, DOC, TN, dissolved oxygen (mg.L-1 and %), pH, pico- and nanoeukaryotes abundances dissolved Cd, Cu, Pb and Zn, and the 17 chemical elements from Table S2) between the different sampling sites (A), sampling date (B), and sampling depth (C). Each symbol corresponds to a distinct site, time and depth point.

Fig. S4 Temporal dynamics of environmental variables of June 2015 in both surface (left panel) and bottom (right panel) seawater samples. Symbols and errors bars in both Dissolved organic carbon and Total nitrogen plots represent average and standard error (n = 3), respectively.

Fig. S5 Dynamics of heterotrophic prokaryotes in surface (A) and bottom (B) seawater at the different sampling stations.

Fig. S6 Box-plot of the Observed OTUs, Chao1, Shannon Simpson and Equitability indices for each sampling sites (i.e. 6ext, MIS, Pt12, Pt15 and 41p) calculated from the 36 remaining samples. Upper and lower lines correspond to the first and third quartile of the distribution of values. The median values are shown with horizontal black wide lines. Outliers are displayed as dots.

Fig. S7 Co-occurrence global network and sub-networks according to MCODE analysis of bacterial taxa and environmental variables derived from the OTU table and showing only statistically significant correlations. Node size correspond to the relative abundance of the OTU. Nodes colors correspond to main bacterioplankton classes. Green and red lines represent positive and negative correlations, respectively.

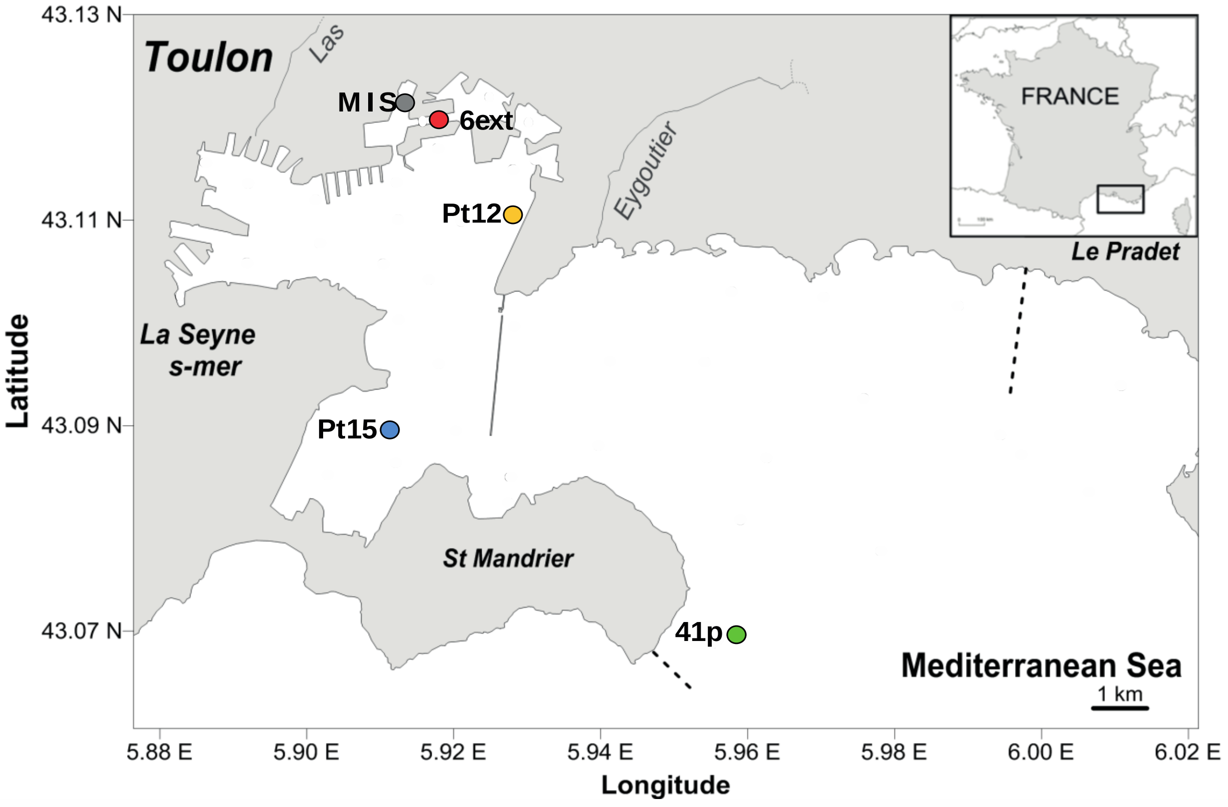

Fig. S1 Map of the studied site Toulon Bay with the location of the sampling sites. Solid and dashed lines represent a seawall and treated sewage submarine outlets, respectively.

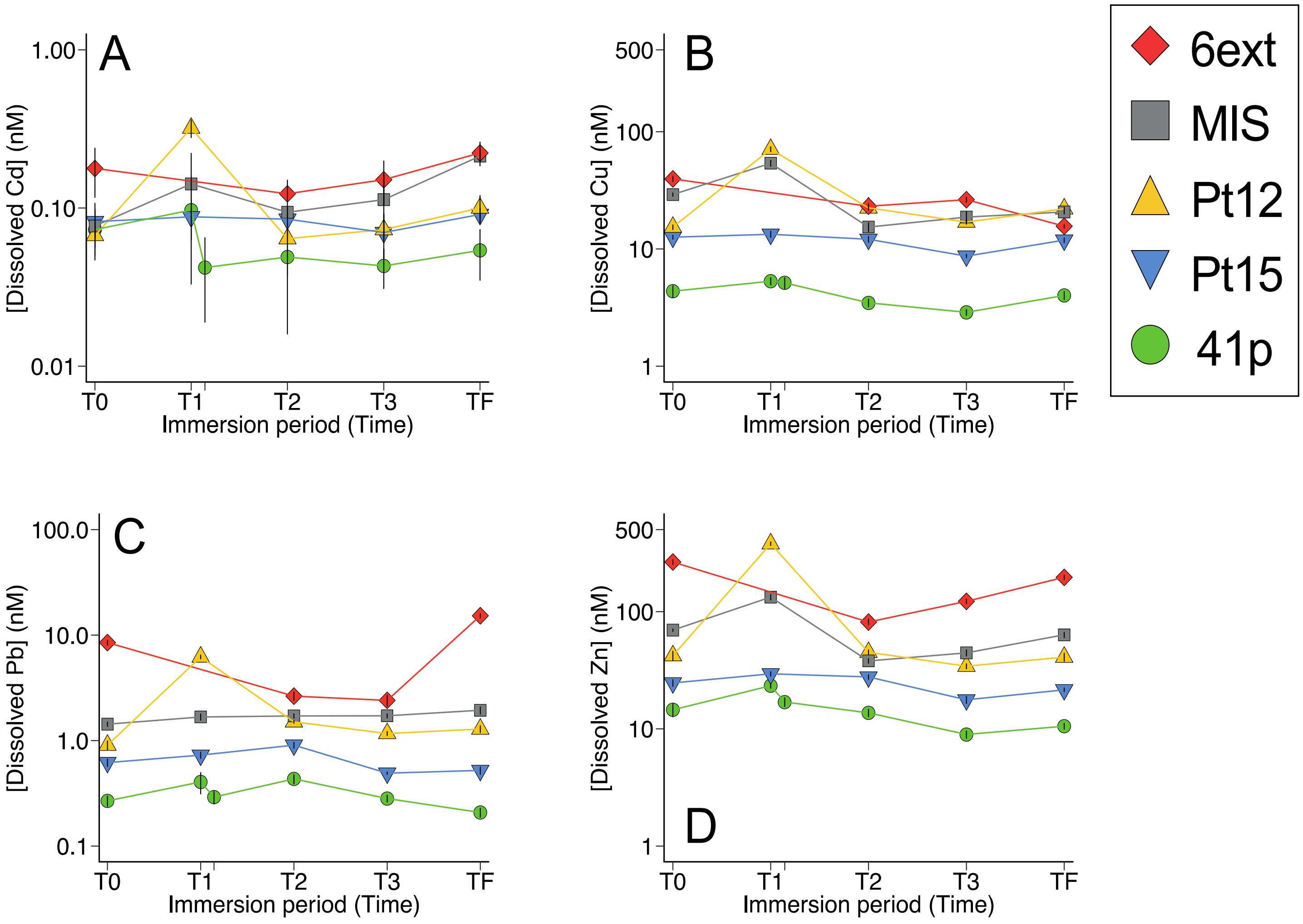

Fig. S2 Dynamics of dissolved trace metals concentrations in bottom seawater at the different sampling stations. Symbols and errors bars represent average and standard error (n = 3), respectively.

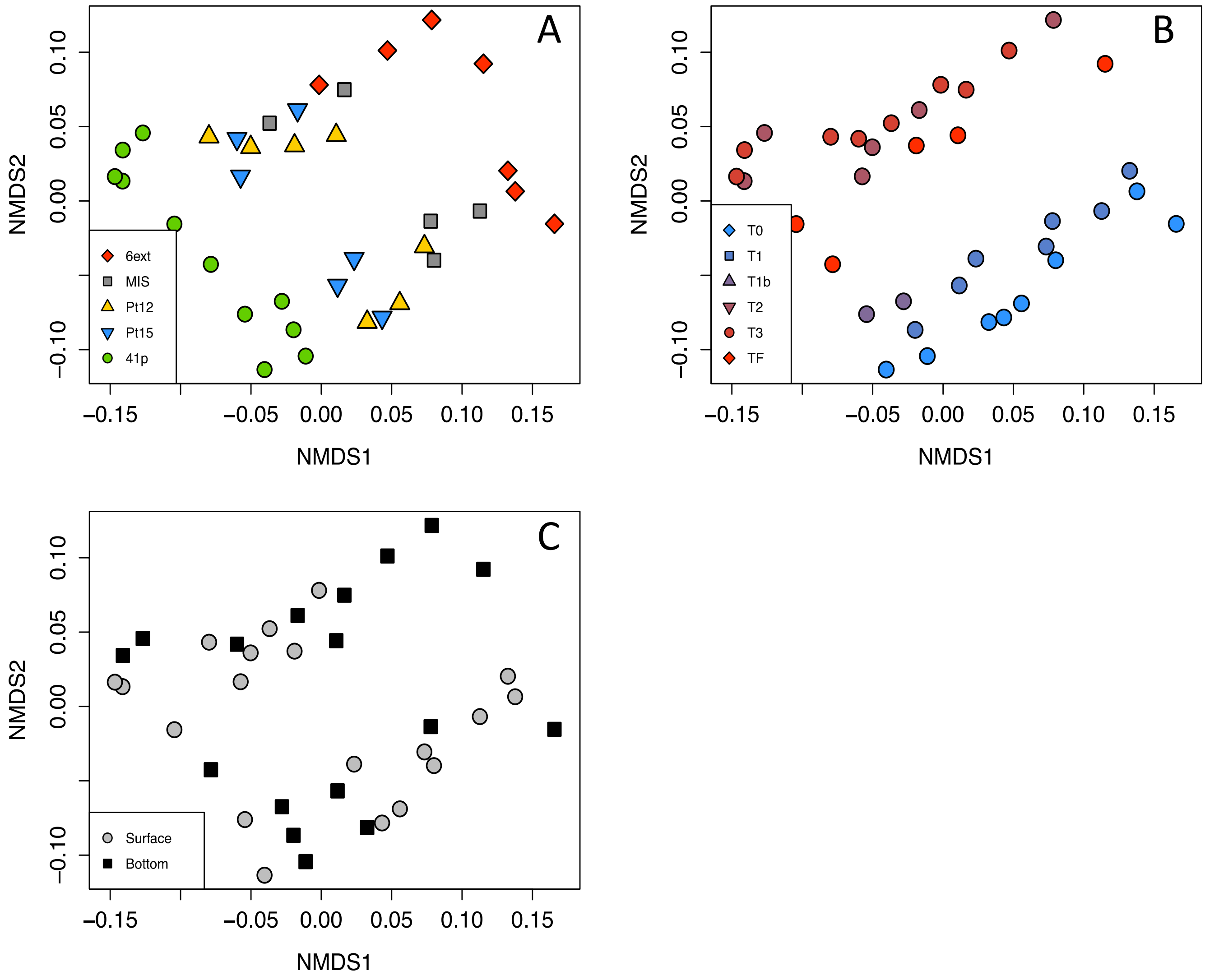

Fig. S3 Nonmetric dimensional scaling (nMDS) ordination based on Bray-Curtis dissimilarity for explanatory variables (temperature, salinity, chlorophyll a, DOC, TN, dissolved oxygen (mg.L-1 and %), pH, pico- and nanoeukaryotes abundances dissolved Cd, Cu, Pb and Zn, and the 17 chemical elements from Table S2) between the different sampling sites (A), sampling date (B), and sampling depth (C). Each symbol corresponds to a distinct site, time and depth point.

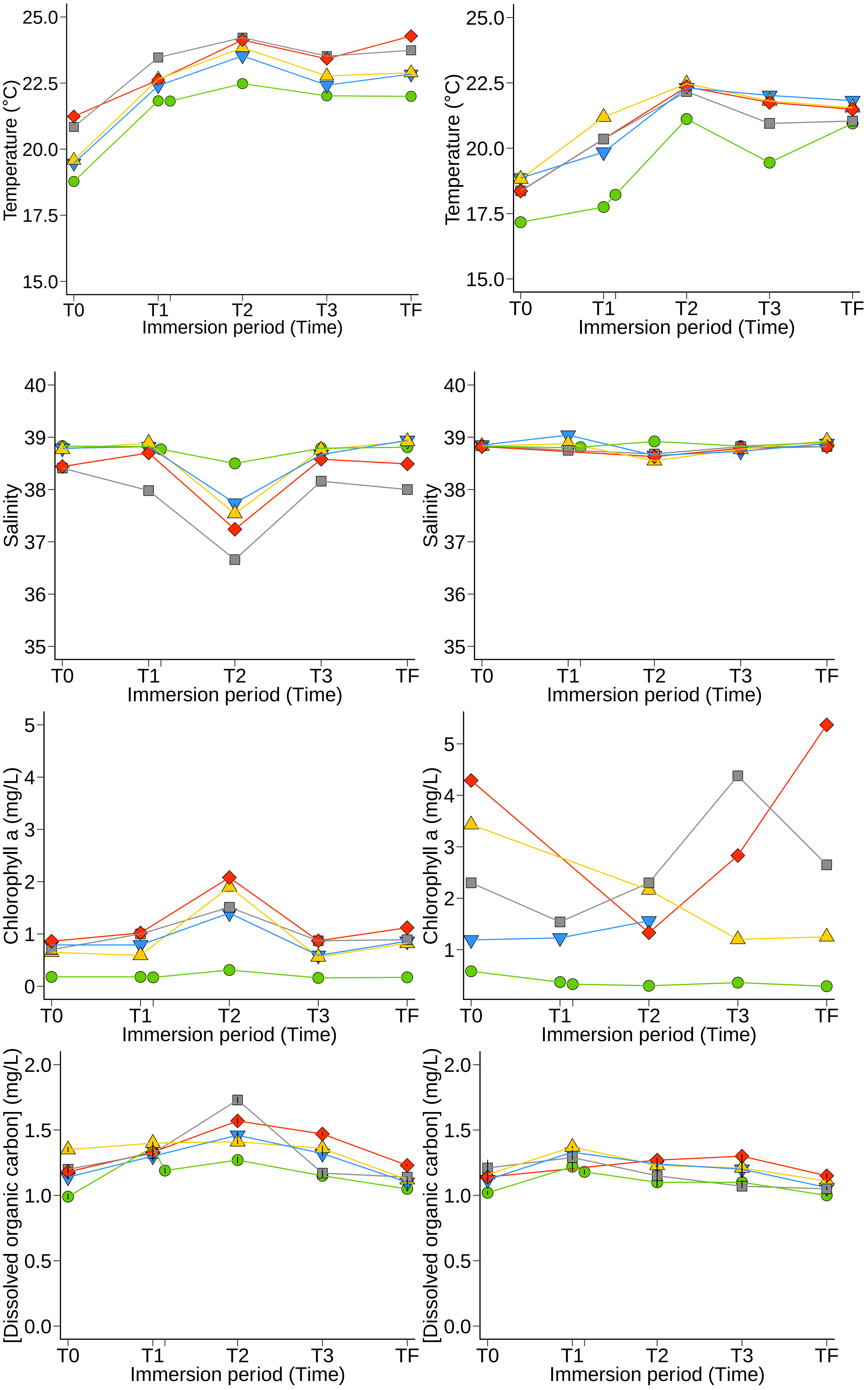

Temperature (°C)
25.0
22.5
20.0
17.5
15.0
T0
T1
T2
T3
TF
Immersion period (Time)
Salinity
40
39
38
37
36
35
Chlorophyll a (mg/L)
5
4
3
2
1
0
[Dissolved organic carbon] (mg/L)
2.0
1.5
1.0
0.5
0.0

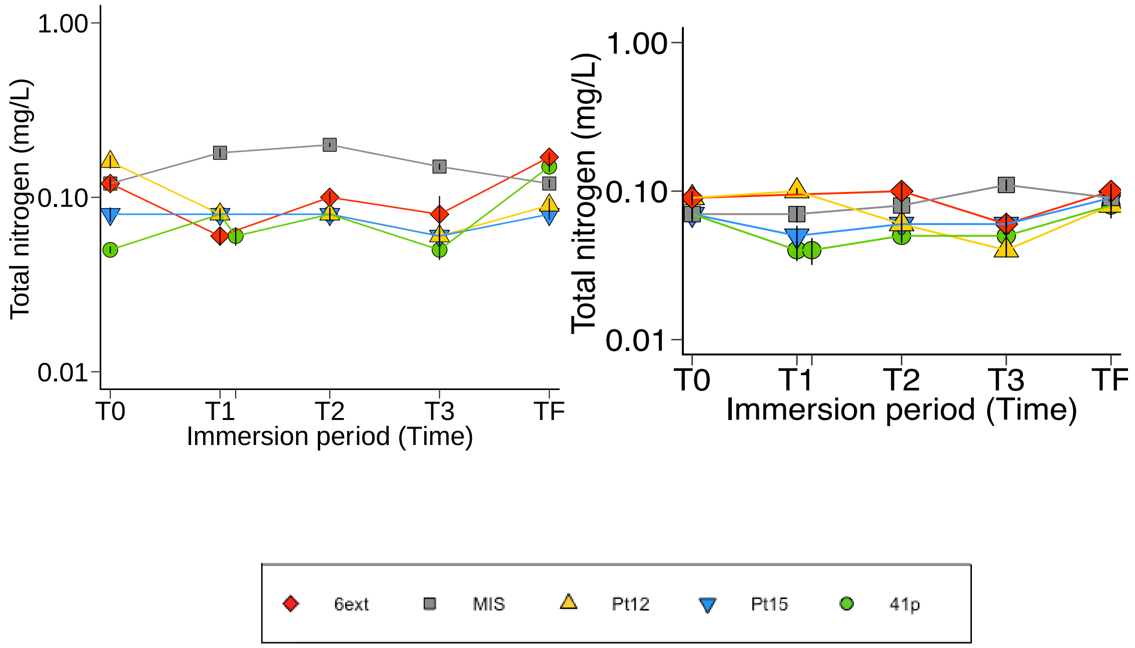

Fig. S4 Temporal dynamics of environmental variables of June 2015 in both surface (left panel) and bottom (right panel) seawater samples. Symbols and errors bars in both Dissolved organic carbon and Total nitrogen plots represent average and standard error (n = 3), respectively.

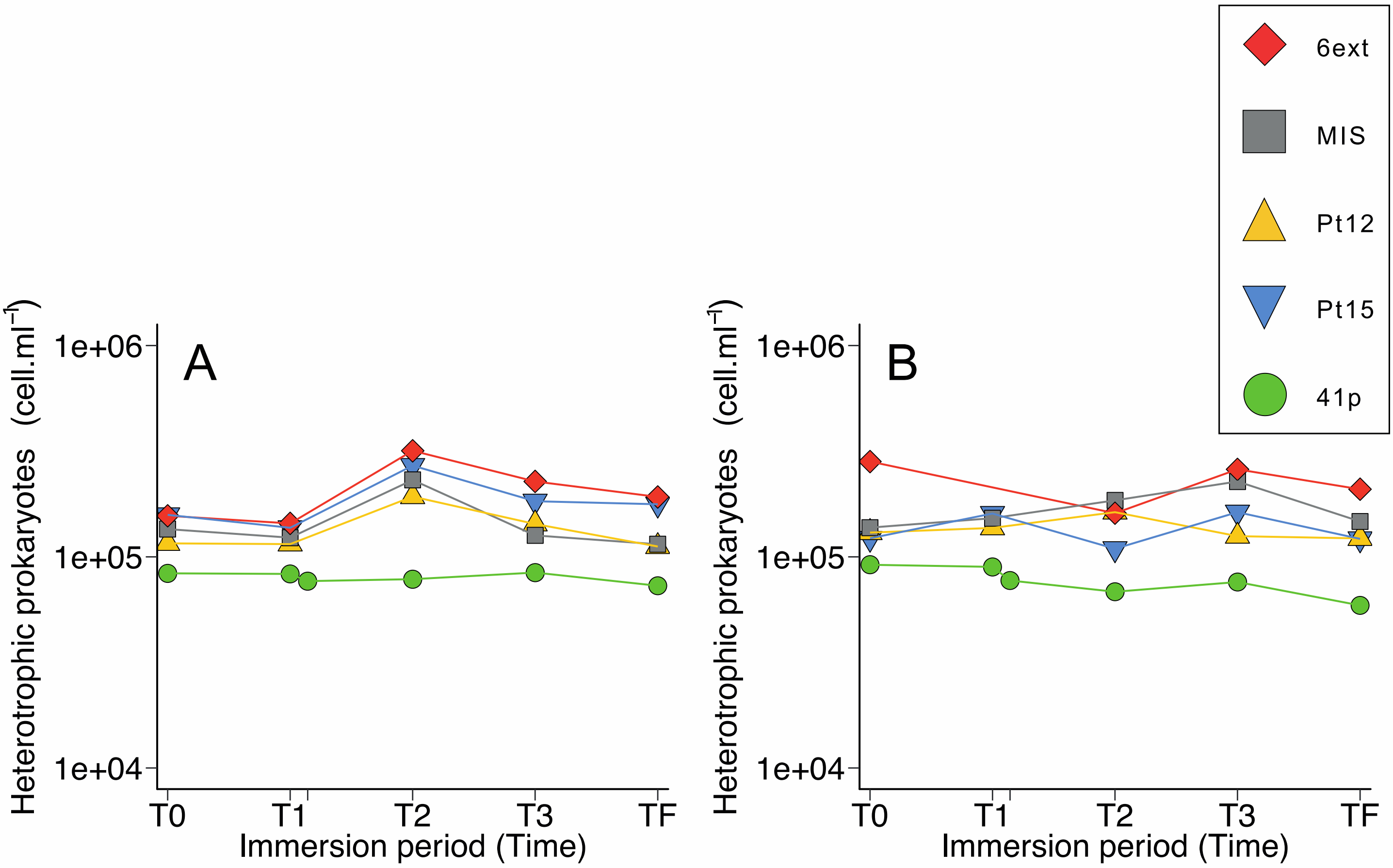

Fig. S5 Dynamics of heterotrophic prokaryotes in surface (A) and bottom (B) seawater at the different sampling stations.

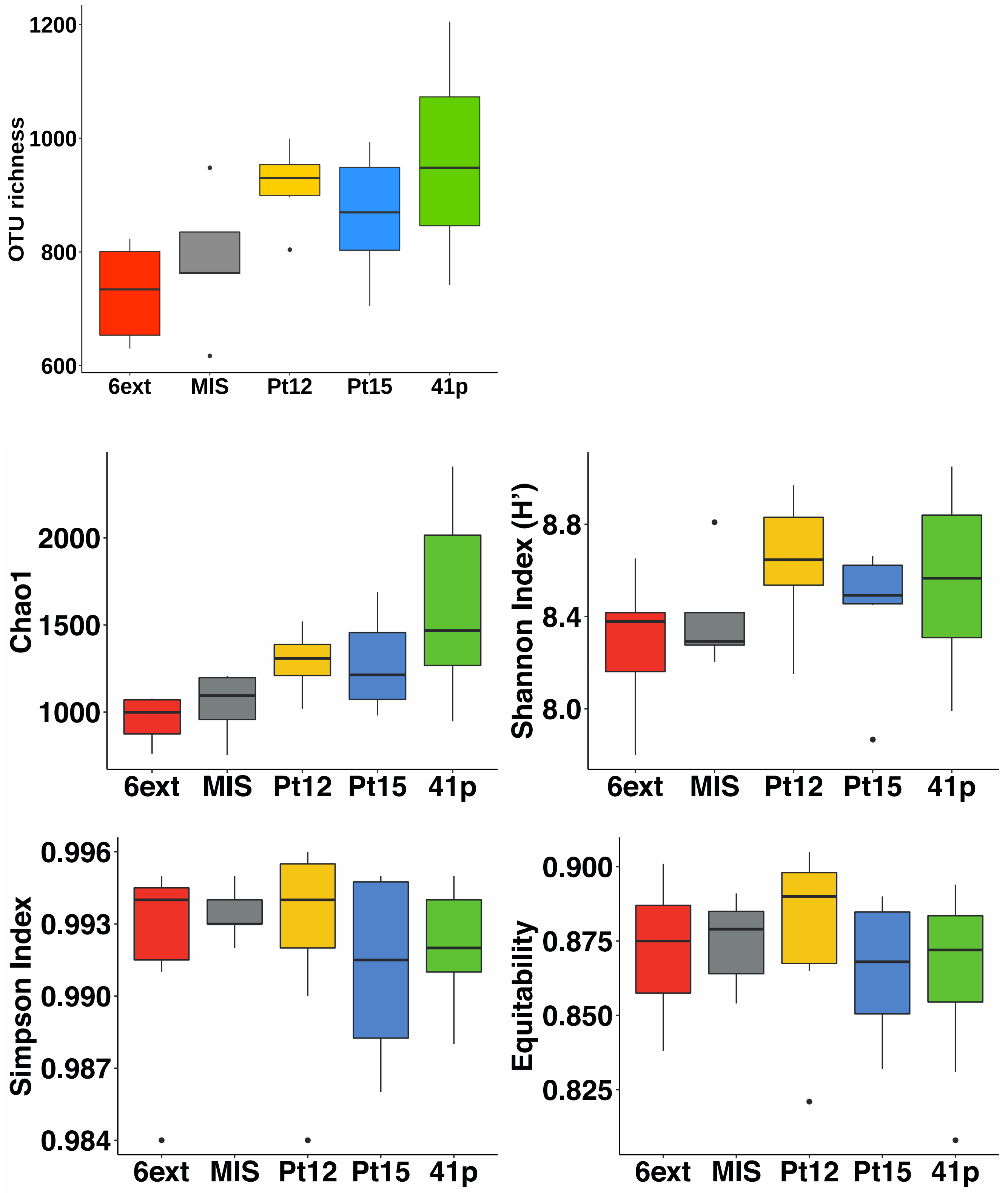

OTU richness
1200
1000
800
600
6ext
MIS
Pt12
Pt15
41p
Chao1
2000
1500
1000
Shannon Index (H')
8.8
8.4
8.0
Simpson Index
0.996
0.993
0.990
0.987
0.984
Equitability
0.900
0.875
0.850
0.825

Fig. S6 Box-plot of the Observed OTUs, Chao1, Shannon Simpson and Equitability indices for each sampling sites (i.e. 6ext, MIS, Pt12, Pt15 and 41p) calculated from the 36 remaining samples. Upper and lower lines correspond to the first and third quartile of the distribution of values. The median values are shown with horizontal black wide lines. Outliers are displayed as dots.

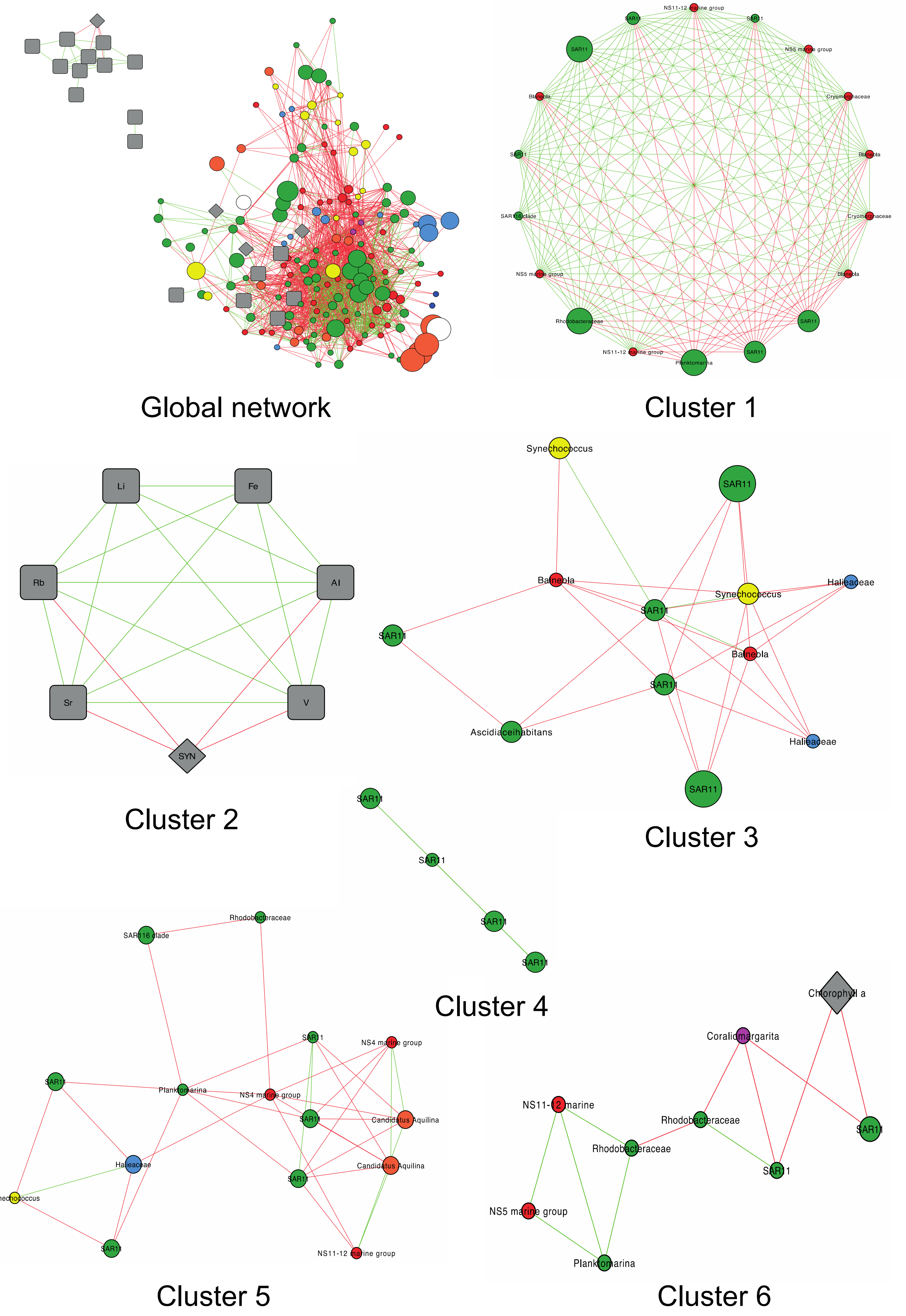

Global network
Cluster 1
NS11-12 marine group
SAR11
SAR11
SAR11
NS5 marine group
Balneola
Cryomorphaceae
SAR11
Balneola
SAR116 clade
Cryomorphaceae
NS5 marine group
Balneola
Rhodobacteraceae
SAR11
NS11-12 marine group
Planktomarina
SAR11
Li
Fe
Rb
Al
Sr
V
SYN
Cluster 2
Synechococcus
SAR11
Balneola
Synechococcus
Haliaceae
SAR11
SAR11
Balneola
SAR11
Ascidiaceihabitans
Haliaceae
SAR11
Cluster 3
SAR11
SAR11
SAR11
SAR11
Cluster 4
Rhodobacteraceae
SAR116 clade
SAR11
NS4 marine group
SAR11
Planktomarina
NS4 marine group
SAR11
Candidatus Aquilina
Haliaceae
Candidatus Aquilina
SAR11
Synechococcus
SAR11
NS11-12 marine group
Cluster 5
Chlorophyll a
Coralidmargarita
NS11-12 marine
Rhodobacteraceae
SAR11
Rhodobacteraceae
SAR11
NS5 marine group
Planktomarina
Cluster 6

Fig. S7 Co-occurrence global network and sub-networks according to MCODE analysis of bacterial taxa and environmental variables derived from the OTU table and showing only statistically significant correlations. Node size correspond to the relative abundance of the OTU. Nodes colors correspond to main bacterioplankton classes. Green and red lines represent positive and negative correlations, respectively.

3. Supplementary Tables

Table S1. Samples characteristics in Toulon Bay.

Table S2. Environmental (biotic and abiotic) characteristics of seawater samples in June 2015. The values represent the mean between surface and bottom samples and the standard deviation for each parameter.

Table S3. Global and pairwise Permutational multivariate analysis of variance (PERMANOVA) examining the effects of the factors "Site", "Date" and "Depth" on the bacterioplankton communities. Key to abbreviations and column headings: D.f, degrees of freedom; MS, mean square; F, F ratio; R^2, coefficient of determination; P, p-value. Probabilities are marked as follows '***' $p<0.001$; '**' $p<0.01$; '*' $p<0.05$.

Table S4. The contribution of particular biomarker to the Bray-Curtis similarity index in 41p-Pt15, 41p-MIS, 41p-6ext, Pt15-MIS, Pt15-6ext and MIS-6ext., using SIMPER (similarity of percentage) analysis. The cut-off for low contribution was set at a cumulative contribution of 90%. P, p-value. Probabilities are marked as follows '***' $p<0.001$; '**' $p<0.01$; '*' $p<0.05$.

Table S5. Results for variation partitioning with the environmental variables, which were selected by forward selection in the redundancy analyses (RDAs) (see Figure 4 in the main manuscript). The contribution (%) of each environmental variable and its significance are given.

Table S6. Network analysis results, showing betweenness centrality (BC), closeness centrality (CC), number of connections (Degree) and relative abundance of keystone OTUs in the global network.

Table S7. Fraction of OTUs not mapped to KEGG organisms (FTU) and percentage of OTUs mapped to KEGG organisms (%)

Table S1. Samples characteristics in Toulon Bay.

Sample Name	Site	Depth	Time	Date	Latitude (°N)	Longitude (°E)	No. of raw amplicons	No. of amplicons after filtering	No. of OTUs	Statistical analyses
41pprof1	41p	Surface	T0	1-Jun-2015	43° 4'21.74"	5°57'21.48"	67550	19242	1045	X
41pprof3	41p	Bottom	T0	1-Jun-2015	43° 4'21.74"	5°57'21.48"	16150	5203	764	X
41pprof5	41p	Surface	T1	8-Jun-2015	43° 4'21.74"	5°57'21.48"	8214	2139	948	
41pprof7	41p	Bottom	T1	8-Jun-2015	43° 4'21.74"	5°57'21.48"	56522	13726	810	X
41pprof9	41p	Surface	T1b	9-Jun-2015	43° 4'21.74"	5°57'21.48"	27834	8457	882	X
41pprof11	41p	Bottom	T1b	9-Jun-2015	43° 4'21.74"	5°57'21.48"	34946	8903	742	X
41pprof13	41p	Surface	T2	15-Jun-2015	43° 4'21.74"	5°57'21.48"	98556	35735	1110	X
41pprof15	41p	Bottom	T2	15-Jun-2015	43° 4'21.74"	5°57'21.48"	48628	14238	1205	X
41pprof17	41p	Surface	T3	22-Jun-2015	43° 4'21.74"	5°57'21.48"	35514	10663	1100	X
41pprof19	41p	Bottom	T3	22-Jun-2015	43° 4'21.74"	5°57'21.48"	40064	12419	909	X
41pprof21	41p	Surface	TF	28-Jun-2015	43° 4'21.74"	5°57'21.48"	30794	7890	965	X
41pprof23	41p	Bottom	TF	28-Jun-2015	43° 4'21.74"	5°57'21.48"	41586	12528	848	X
15prof1	Pt15	Surface	T0	1-Jun-2015	43° 5'24.82"	5°54'39.05"	15760	4349	788	X
15prof3	Pt15	Bottom	T0	1-Jun-2015	43° 5'24.82"	5°54'39.05"	12240	2908	993	
15prof5	Pt15	Surface	T1	8-Jun-2015	43° 5'24.82"	5°54'39.05"	24982	5941	705	X
15prof7	Pt15	Bottom	T1	8-Jun-2015	43° 5'24.82"	5°54'39.05"	45838	8534	891	X
15prof9	Pt15	Surface	T2	15-Jun-2015	43° 5'24.82"	5°54'39.05"	47776	13051	968	X
15prof11	Pt15	Bottom	T2	15-Jun-2015	43° 5'24.82"	5°54'39.05"	45218	9660	804	X
15prof13	Pt15	Surface	T3	22-Jun-2015	43° 5'24.82"	5°54'39.05"	32980	6527	999	X
15prof15	Pt15	Bottom	T3	22-Jun-2015	43° 5'24.82"	5°54'39.05"	7124	1557	903	
15prof17	Pt15	Surface	TF	28-Jun-2015	43° 5'24.82"	5°54'39.05"	8202	1928	896	
15prof19	Pt15	Bottom	TF	28-Jun-2015	43° 5'24.82"	5°54'39.05"	9788	2584	936	
12prof1	Pt12	Surface	T0	1-Jun-2015	43° 6'34.96"	5°55'41.09"	21820	4633	971	X
12prof3	Pt12	Bottom	T0	1-Jun-2015	43° 6'34.96"	5°55'41.09"	30610	6160	930	X
12prof5	Pt12	Surface	T1	8-Jun-2015	43° 6'34.96"	5°55'41.09"	38604	7060	762	X
12prof7	Pt12	Bottom	T1	8-Jun-2015	43° 6'34.96"	5°55'41.09"	8746	1266	763	
12prof9	Pt12	Surface	T2	15-Jun-2015	43° 6'34.96"	5°55'41.09"	16898	2813	835	
12prof11	Pt12	Bottom	T2	15-Jun-2015	43° 6'34.96"	5°55'41.09"	23846	4125	948	X
12prof13	Pt12	Surface	T3	22-Jun-2015	43° 6'34.96"	5°55'41.09"	17414	2368	617	
12prof15	Pt12	Bottom	T3	22-Jun-2015	43° 6'34.96"	5°55'41.09"	33336	7427	800	X
12prof17	Pt12	Surface	TF	28-Jun-2015	43° 6'34.96"	5°55'41.09"	55852	7208	635	X
12prof19	Pt12	Bottom	TF	28-Jun-2015	43° 6'34.96"	5°55'41.09"	28124	7004	630	X
MISprof1	MIS	Surface	T0	1-Jun-2015	43° 7'9.37"	5°54'40.07"	19036	4044	734	X
MISprof3	MIS	Bottom	T0	1-Jun-2015	43° 7'9.37"	5°54'40.07"	13550	2828	801	
MISprof5	MIS	Surface	T1	8-Jun-2015	43° 7'9.37"	5°54'40.07"	25806	5595	823	X
MISprof7	MIS	Bottom	T1	8-Jun-2015	43° 7'9.37"	5°54'40.07"	27530	4296	672	X
MISprof9	MIS	Surface	T2	15-Jun-2015	43° 7'9.37"	5°54'40.07"	16944	3730		
MISprof11	MIS	Bottom	T2	15-Jun-2015	43° 7'9.37"	5°54'40.07"	14946	2977		
MISprof13	MIS	Surface	T3	22-Jun-2015	43° 7'9.37"	5°54'40.07"	20490	4057		X
MISprof15	MIS	Bottom	T3	22-Jun-2015	43° 7'9.37"	5°54'40.07"	21712	4650		X

MISprof17	MIS	Surface	TF	28-Jun-2015	43° 7'9.37"	5°54'40.07"	17270	3913	
MISprof19	MIS	Bottom	TF	28-Jun-2015	43° 7'9.37"	5°54'40.07"	21438	3350	
6extprof1	6ext	Surface	T0	1-Jun-2015	43° 7'8.65"	5°54'57.31"	22718	4846	X
6extprof3	6ext	Bottom	T0	1-Jun-2015	43° 7'8.65"	5°54'57.31"	32860	7517	X
6extprof5	6ext	Surface	T1	8-Jun-2015	43° 7'8.65"	5°54'57.31"	30190	5032	X
6extprof7	6ext	Bottom	T1	8-Jun-2015	43° 7'8.65"	5°54'57.31"	14116	4170	X
6extprof9	6ext	Surface	T2	15-Jun-2015	43° 7'8.65"	5°54'57.31"	16930	4044	X
6extprof11	6ext	Bottom	T2	15-Jun-2015	43° 7'8.65"	5°54'57.31"	44004	3948	
6extprof13	6ext	Surface	T3	22-Jun-2015	43° 7'8.65"	5°54'57.31"	20680	4549	X
6extprof15	6ext	Bottom	T3	22-Jun-2015	43° 7'8.65"	5°54'57.31"	9016	2552	
6extprof17	6ext	Surface	TF	28-Jun-2015	43° 7'8.65"	5°54'57.31"	54180	11316	X
6extprof19	6ext	Bottom	TF	28-Jun-2015	43° 7'8.65"	5°54'57.31"	13856	3349	

Table S2. Environmental (biotic and abiotic) characteristics of seawater samples in June 2015. The values represent the mean between surface and bottom samples and the standard deviation for each parameter.

Site	Date	Al (nM)	As (nM)	Ba (nM)	Be (nM)	Cs (nM)	Cr (nM)	Fe (nM)	Li (nM)	Mn (nM)
41p	T0	9358 ± 405	28 ± 0,5	59 ± 0,20	23 ± 5,2	2,6 ± 0,0	14 ± 1,9	116 ± 9,5	33167 ± 1818	17 ± 0,60
	T1	8918 ± 100	23 ± 2,5	61 ± 1,7	24 ± 7,2	2,7 ± 0,05	7,8 ± 0,45	98 ± 9,7	30461 ± 382	17 ± 2,2
	T1b	8330 ± 236	25 ± 2,3	63 ± 0,35	39 ± 6,5	2,6 ± 0,0	6,4 ± 0,90	88 ± 1,4	28587 ± 594	15 ± 0,60
	T2	52 ± 18	23 ± 0,35	59 ± 1,6	44 ± 9,8	2,5 ± 0,10	3,9 ± 1,9	15 ± 0,09	27782 ± 379	11 ± 2,2
	T3	193 ± 26	30 ± 3,6	57 ± 0,85	53 ± 1,3	2,5 ± 0,05	4,6 ± 0,55	13 ± 3,1	26165 ± 29	10 ± 1,2
	TF	2068 ± 87	28 ± 2,3	57 ± 0,45	89 ± 0,65	2,4 ± 0,0	6,3 ± 0,8	55 ± 21	27020 ± 147	14 ± 0,09
Pt15	T0	11044 ± 89	27 ± 2,7	60 ± 0,05	41 ± 2,0	2,6 ± 0,0	11 ± 1,2	155 ± 9,4	37593 ± 537	31 ± 2,7
	T1	8594 ± 196	30 ± 0,25	64 ± 0,95	36 ± 5,2	2,6 ± 0,0	7,9 ± 0,55	103 ± 14	28962 ± 147	28 ± 2,6
	T2	137 ± 46	24 ± 0,25	59 ± 2,05	36 ± 10	2,4 ± 0,0	5,3 ± 0,0	45 ± 15	27022 ± 56	27 ± 6,3
	T3	252 ± 29	29 ± 4.0	59 ± 0,05	44 ± 3,9	2,5 ± 0,0	5,7 ± 0,75	33 ± 6,6	26242 ± 104	17 ± 1,4
	TF	2228 ± 51	26 ± 2,0	60 ± 0,05	72 ± 19	2,6 ± 0,0	5,2 ± 0,80	158 ± 110	27834 ± 112	17 ± 1,3
Pt12	T0	11007 ± 97	25 ± 3,3	59 ± 0,75	42 ± 5,2	2,6 ± 0,05	11 ± 0,20	133 ± 2,7	36473 ± 15	25 ± 1,9
	T1	9070 ± 652	27 ± 2,4	70 ± 5,4	33 ± 0,0	2,6 ± 0,0	5,8 ± 0,25	108 ± 6,0	29454 ± 279	41 ± 11
	T2	163 ± 31	24 ± 1,9	59 ± 1,2	42 ± 0,0	2,4 ± 0,0	5,6 ± 0,25	21 ± 7,5	26666 ± 239	29 ± 3,2
	T3	344 ± 12	24 ± 7,05	59 ± 0,0	64 ± 5,2	2,6 ± 0,05	5,2 ± 0,6	20 ± 1,7	27049 ± 347	18 ± 0,65
	TF	2050± 500	21 ± 3,0	58 ± 0,95	65 ± 13	2,5 ± 0,0	9,6 ± 3,0	41 ± 7,9	25734 ± 1912	18 ± 1,05
MIS	T0	9663 ± 195	26 ± 1,7	63 ± 0,90	34 ± 1,3	2,6 ± 0,05	10 ± 0,45	110 ± 2,0	33726 ± 409	25 ± 1,2
	T1	8654 ± 161	20 ± 1,9	65 ± 2,7	46 ± 0,0	2,6 ± 0,0	6,8 ± 1,7	104 ± 2,2	28456 ± 505	30 ± 0,40
	T2	136 ± 50	24 ± 0,80	62 ± 3,3	50 ± 3,3	2,4 ± 0,10	5,05 ± 0,45	28 ± 9,3	26153 ± 393	33 ± 3,0
	T3	356 ± 41	28 ± 3,3	59 ± 1,3	64 ± 0,65	2,5 ± 0,05	5,5 ± 0,35	29 ± 3,0	27067 ± 419	20 ± 0,50
	TF	1940 ± 87	28 ± 1,2	59 ± 0,20	83 ± 22	2,5 ± 0,05	8,2 ± 1,3	50 ± 0,75	24853 ± 610	17 ± 0,0
6ext	T0	10154 ± 571	26 ± 1,2	70 ± 1,6	31 ± 2,0	2,6 ± 0,0	6,4 ± 0,2	120 ± 17	31818 ± 217	44 ± 13
	T1	8392 ± 0,0	27 ± 0,0	74 ± 0,0	47 ± 0,0	2,7 ± 0,0	6,6 ± 0,0	111 ± 0,0	29390 ± 0,0	34 ± 0,0
	T2	193 ± 32	30 ± 2,0	65 ± 2,7	46 ± 3,3	2,5 ± 0,05	4,8 ± 0,15	33 ± 6,9	25905 ± 669	41 ± 5,1
	T3	327 ± 9,6	27 ± 7,1	63 ± 1,7	79 ± 8,5	2,5 ± 0,0	4,5 ± 0,10	42 ± 0,5	27231 ± 640	32 ± 9,5
	TF	2614 ± 51	33 ± 0,85	73 ± 6,1	107 ± 1,3	2,6 ± 0,05	6,2 ± 1,8	63 ± 7,0	26565 ± 77	37 ± 11

Table S2. Continued

Site	Date	Mo95	Rb	Sb	Sn	Sr	Ti	U	V
		(nM)	(nM)	(nM)	(nM)	(nM)	(nM)	(nM)	(nM)
41p	T0	152 ± 2,0	1529 ± 27	3,6 ± 0,05	2,0 ± 0,45	109807 ± 1597	25 ± 1,5	13 ± 0,75	47 ± 1,9
	T1	159 ± 3,0	1502 ± 8,2	3,4 ± 0,20	2,0 ± 0,05	108072 ± 3623	23 ± 1,0	16 ± 0,35	46 ± 1,7
	T1b	171 ± 0,55	1464 ± 10	3,3 ± 0,20	2,2 ± 0,10	106015 ± 702	22 ± 1,4	17 ± 0,0	45 ± 0,101
	T2	164 ± 11	1461 ± 14	3,6 ± 0,05	1,8 ± 0,15	104642 ± 1296	0,80 ± 0,0	16 ± 1,3	41 ± 0,30
	T3	1601 ± 0,65	1450 ± 1,0	3,7 ± 0,09	1,4 ± 0,0	103385 ± 474	3,2 ± 1,1	16 ± 0,0	39 ± 1,5
	TF	168 ± 0,05	1453 ± 6,0	3,9 ± 0,10	1,2 ± 0,0	103734 ± 75	6,2 ± 0,40	16 ± 0,0	43 ± 1,8
Pt15	T0	159 ± 1,7	1593 ± 9,8	3,8 ± 0,20	2,35 ± 0,05	113367 ± 718	29 ± 2,9	12 ± 0,15	50 ± 0,0
	T1	164 ± 1,0	1467 ± 4,9	3,5 ± 0,0	2,1 ± 0,0	104931 ± 701	23 ± 1,8	17 ± 0,10	45 ± 1,4
	T2	151 ± 0,55	1429 ± 11	3,7 ± 0,0	1,7 ± 0,0	102450 ± 969	2,1 ± 0,0	15 ± 0,10	40 ± 0,0
	T3	162 ± 0,50	1439 ± 3,9	3,9 ± 0,10	1,35 ± 0,05	104415 ± 351	2,3 ± 1,7	16 ± 0,05	40 ± 2,2
	TF	172 ± 1,6	1457 ± 9,3	5,5 ± 0,05	1,2 ± 0,0	104481 ± 612	8,3 ± 1,3	16 ± 0,10	44 ± 0,20
Pt12	T0	156 ± 0,35	1556 ± 0,40	3,7 ± 0,05	2,2 ± 0,0	111328 ± 778	32 ± 0,50	12 ± 0,35	49 ± 0,40
	T1	163 ± 1,2	1479 ± 2,3	4,8 ± 1,2	3,6 ± 1,3	106465 ± 396	25 ± 0,60	17 ± 0,0	44 ± 2,2
	T2	153 ± 2,4	1420 ± 16	3,8 ± 0,10	1,65 ± 0,05	101902 ± 383	0,60 ± 0,0	15 ± 0,15	39 ± 0,40
	T3	164 ± 2,2	1441 ± 2,5	4,0 ± 0,15	1,3 ± 0,0	103906 ± 8,6	3,1 ± 1,2	16 ± 0,0	40 ± 1,7
	TF	165 ± 5,8	1428 ± 41	5,7 ± 0,35	1 ± 0,0	102253 ± 1402	5,4 ± 1,3	16 ± 0,35	39 ± 1,7
MIS	T0	154 ± 1,0	1524 ± 2,0	3,5 ± 0,10	1,95 ± 0,05	109185 ± 607	33 ± 1,6	14 ± 0,25	47 ± 0,40
	T1	167 ± 3,2	1459 ± 15	3,8 ± 0,10	2,3 ± 0,10	105079 ± 863	27 ± 1,5	17 ± 0,15	44 ± 1,0
	T2	155 ± 2,3	1424 ± 21	4,1 ± 0,35	1,6 ± 0,0	102697 ± 1857	0,80 ± 0,0	15 ± 0,25	38 ± 0,20
	T3	165 ± 0,25	1432 ± 11	4,2 ± 0,05	1,5 ± 0,0	102172 ± 1461,05	1,8 ± 0,95	16 ± 0,10	40 ± 0,80
	TF	163 ± 1,0	1405 ± 13	5,7 ± 0,10	1,05 ± 0,05	101164 ± 810	9,4 ± 1,6	16 ± 0,15	39 ± 0,70
6ext	T0	157 ± 0,40	1505 ± 2,2	4,9 ± 0,20	2,35 ± 0,05	107618 ± 321	30 ± 3,6	15 ± 0,25	46 ± 1,6
	T1	173 ± 0,0	1489 ± 0,0	4,8 ± 0,0	2,4 ± 0,0	107221 ± 0,0	27 ± 0,0	17 ± 0,0	44 ± 0,0
	T2	155 ± 2,7	1418 ± 37	4,7 ± 0,30	1,7 ± 0,0	102095 ± 2733	1,4 ± 0,35	15 ± 0,40	38 ± 1,7
	T3	167 ± 2,8	1450 ± 19	5,7 ± 0,20	1,55 ± 0,15	103881 ± 490	4,5 ± 1,2	16 ± 0,30	40 ± 1,7
	TF	168 ± 0,75	1440 ± 12	7,6 ± 1,0	1,55 ± 0,25	103208 ± 28	9,8 ± 3,4	16 ± 0,20	44 ± 1,0

Table S2. Continued

Site	Date	Dissolved oxygen	Dissolved oxygen	pH	Picoeukaryote	Nanoeukaryote
		(mg.L^{-1})	(%)	-	Cell.mL^{-1}	Cell.mL^{-1}
41p	T0	114 ± 0.15	8.6 ± 0.12	8.7 ± 0.01	2652 ± 244	1616 ± 26
	T1	117 ± 1.5	8.6 ± 0.22	8.6 ± 0.02	52 ± 28	76 ± 26
	T1b	119 ± 1.6	8.7 ± 0.40	8.6 ± 0.01	728 ± 500	1398 ± 556
	T2	110 ± 1.8	7.8 ± 0.21	8.7 ± 0.02	1236 ± 736	1357 ± 307
	T3	112 ± 2.8	8.0 ± 0.39	7.9 ± 0.10	492 ± 132	1049 ± 107
	TF	109 ± 0.70	7.8 ± 0.12	7.9 ± 0.02	286 ± 76	441 ± 29
Pt15	T0	117 ± 3.6	8.3 ± 0.22	8.7 ± 0.01	6737 ± 1995	4635 ± 1409
	T1	116 ± 2.6	8.3 ± 0.0	8.7 ± 0.04	327 ± 97	313 ± 39
	T2	112 ± 1.2	7.8 ± 0.03	8.7 ± 0.01	14068 ± 8138	4763 ± 1653
	T3	114 ± 1.7	8.0 ± 0.10	8.0 ± 0.02	2069 ± 261	2475 ± 185
	TF	113 ± 1.3	7.9 ± 0.02	7.9 ± 0.01	3420 ± NA	2352 ± NA
Pt12	T0	117 ± 0.65	NA	8.7 ± 0.01	3750 ± 372	3049 ± 139
	T1	117 ± 1.1	8.3 ± 0.19	8.7 ± 0.01	505 ± 47	644 ± 320
	T2	113 ± 2.6	7.8 ± 0.11	8.7 ± 0.02	12033 ± 3589	6821 ± 2283
	T3	114 ± 0.19	8.0 ± 0.08	8.0 ± 0.02	2675 ± 577	2889 ± 39
	TF	116 ± 0.15	8.1 ± 0.09	8.0 ± 0.03	2967 ± 1123	2678 ± 460
MIS	T0	123 ± 1.7	NA	8.6 ± 0.03	5165 ± 729	4246 ± 290
	T1	122 ± 1.2	8.6 ± 0.14	8.7 ± 0.04	6690 ± 3656	5429 ± 963
	T2	109 ± 12	7.6 ± 0.73	8.7 ± 0.04	14735 ± 6003	8700 ± 3826
	T3	111 ± 5.4	7.8 ± 0.22	7.9 ± 0.03	8153 ± 1749	4078 ± 796
	TF	117 ± 1.6	8.2 ± 0.07	7.9 ± 0.02	11351 ± 3131	5537 ± 471
6ext	T0	113 ± 8.4	8.3 ± 0.40	8.6 ± 0.04	15247 ± 4973	6236 ± 624
	T1	NA	NA	NA	926 ± NA	2230 ± NA
	T2	106 ± 15	7.3 ± 0.93	8.7 ± 0.01	12863 ± 6431	7486 ± 4612
	T3	108 ± 5.9	7.6 ± 0.30	7.9 ± 0.03	8403 ± 1285	3444 ± 330
	TF	113 ± 8.2	7.8 ± 0.39	7.9 ± 0.10	16378 ± 3204	5666 ± 1492

Table S3. Global and pairwise Permutational multivariate analysis of variance (PERMANOVA) examining the effects of the factors "Site", "Date" and "Depth" on the bacterioplankton communities. Key to abbreviations and column headings: D.f, degrees of freedom; MS, mean square; F, F ratio; R^2, coefficient of determination; P, p-value. Probabilities are marked as follows '***' p<0.001; '**' p<0.01; '*' p<0.05.

Source of variation	D.f	MS	F	R^2	P	Significance
**Site**	**4**	**0.146172**	**7.1909**	**0.46071**	**0.001**	***
**Date**	**5**	**0.043509**	**2.1404**	**0.17142**	**0.024**	*
Depth	1	0.002537	0.1248	0.00200	0.983	
Site:Date	13	0.016955	0.8341	0.17367	0.696	
Residuals	12	0.020327	0.19221			
Total	35	1.00000				

	41p	6ext	MIS	Pt12
6ext	**0.0050** ***	-	-	-
MIS	**0.0050** ***	0.9270	-	-
Pt12	**0.0067** ***	**0.0280** *	0.1714	-
Pt15	**0.0125** *	**0.0483** *	0.1725	0.3522

Table S4. The contribution of particular biomarker to the Bray-Curtis similarity index in 41p-Pt15, 41p-MIS, 41p-6ext, Pt15-MIS, Pt15-6ext and MIS-6ext., using SIMPER (similarity of percentage) analysis. The cut-off for low contribution was set at a cumulative contribution of 90%. P, p-value. Probabilities are marked as follows '***' p<0.001; '**' p<0.01; '*' p<0.05.

**41p - Pt15**				
Biomarker		Contribution	Cumulative	P
Higher taxonomic rank	Phylum / Class	%	%	-
PS1 clade	Alphaproteobacteria	1.81	32.42	0.004 **
PS1 clade uncultured bacterium	Alphaproteobacteria	1.72	35.32	0.007 **
OM43 clade	Betaproteobacteria	1.69	38.17	0.001 ***
Methylophilaceae	Betaproteobacteria	1.67	40.99	0.002 **
Methylophilales	Betaproteobacteria	1.67	43.80	0.002 **
Betaproteobacteria	Proteobacteria	1.50	48.99	0.001 ***
NS7 marine group Other	Flavobacteriia	1.49	51.49	0.004 **
NS7 marine group	Flavobacteriia	1.49	54.00	0.004 **
SAR406 clade uncultured bacterium	Marinimicrobia	1.43	56.41	0.003 **
SAR406 clade	Marinimicrobia	1.43	58.83	0.003 **
S25_593 uncultured bacterium	Alphaproteobacteria	1.27	60.98	0.003 **
S25_593	Alphaproteobacteria	1.27	63.12	0.003 **
Rhizobiales	Alphaproteobacteria	1.19	67.24	0.006 **
Surface 4	Alphaproteobacteria	1.15	69.17	0.025 *
Flammeovirgaceae uncultured	Cytophagia	1.13	71.08	0.008 **
Rickettsiales	Alphaproteobacteria	1.06	78.35	0.001 ***
SAR116 clade	Alphaproteobacteria	1.05	80.12	0.002 **

**41p - MIS**				
Biomarker		Contribution	Cumulative	P
Higher taxonomic rank	Phylum / Class	%	%	-
Candidatus Aquiluna	Actinobacteria	6.68	9.61	0.002 **
Microbacteriaceae	Actinobacteria	5.55	17.58	0.002 **
Micrococcales	Actinobacteria	5.53	25.54	0.002 **
Actinobacteria	Actinobacteria	5.08	32.85	0.003 **
PS1 clade	Alphaproteobacteria	2.11	42.58	0.003 **
PS1 clade uncultured bacterium	Alphaproteobacteria	1.91	45.33	0.017 *
NS7 marine group Other	Flavobacteriia	1.79	47.89	0.001 ***
NS7 marine group	Flavobacteriia	1.79	50.46	0.001 ***
Surface 4	Alphaproteobacteria	1.47	54.70	0.003 **
SAR406 clade uncultured bacterium	Marinimicrobia	1.47	56.81	0.009 **
SAR406 clade	Marinimicrobia	1.47	58.92	0.009 **
Candidatus Actinomarina	Acidimicrobiia	1.46	61.02	0.012 *

OM1 clade	Acidimicrobiia	1.44	63.09	0.012 *
S25_593 uncultured bacterium	Alphaproteobacteria	1.38	65.07	0.006 **
S25_593	Alphaproteobacteria	1.38	67.04	0.006 **
Acidimicrobiales	Acidimicrobiia	1.37	69.01	0.012 *
Acidimicrobiia	Actinobacteria	1.37	70.97	0.012 *
Rhizobiales	Alphaproteobacteria	1.36	72.92	0.002 **
SAR116 clade	Alphaproteobacteria	1.30	74.80	0.001 ***
Rickettsiales	Alphaproteobacteria	1.29	76.65	0.001 ***
Thiothrix	Gammaproteobacteria	1.26	78.46	0.024 *
Thiotrichaceae	Gammaproteobacteria	1.26	80.26	0.024 *
Thiotrichales	Gammaproteobacteria	1.26	82.07	0.024 *
Flammeovirgaceae uncultured	Cytophagia	1.16	83.74	0.035 *
SAR116 clade uncultured bacterium	Alphaproteobacteria	1.00	89.86	0.001 ***

**41p - 6ext**				
Biomarker		Contribution	Cumulative	P
Higher taxonomic rank	Phylum / Class	%	%	-
Candidatus Aquiluna	Actinobacteria	5.75	7.74	0.008 **
Microbacteriaceae	Actinobacteria	4.79	14.19	0.010 **
Micrococcales	Actinobacteria	4.78	20.63	0.010 **
Planktomarina	Alphaproteobacteria	4.66	26.90	0.001 ***
Actinobacteria	Actinobacteria	4.35	32.75	0.017 *
Tenacibaculum	Flavobacteriia	3.25	37.14	0.001 ***
PS1 clade	Alphaproteobacteria	2.01	39.85	0.002 **
NS7 marine group Other	Flavobacteriia	1.90	42.40	0.001 ***
NS7 marine group	Flavobacteriia	1.90	44.96	0.001 ***
PS1 clade uncultured bacterium	Alphaproteobacteria	1.83	47.42	0.007 **
Formosa	Flavobacteriia	1.67	49.67	0.001 ***
Candidatus Actinomarina	Acidimicrobiia	1.65	51.89	0.001 ***
OM1 clade	Acidimicrobiia	1.63	54.08	0.001 ***
Surface 4 uncultured bacterium	Alphaproteobacteria	1.58	56.21	0.003 **
Acidimicrobiales	Acidimicrobiia	1.53	58.27	0.001 ***
Acidimicrobiia	Actinobacteria	1.53	60.33	0.001 ***
Surface 4	Alphaproteobacteria	1.52	62.37	0.001 ***
S25_593 uncultured bacterium	Alphaproteobacteria	1.44	64.31	0.001 ***
S25_593	Alphaproteobacteria	1.44	66.24	0.001 ***
OM43 clade	Betaproteobacteria	1.40	68.13	0.037 *
Methylophilaceae	Betaproteobacteria	1.38	70.00	0.049 *
Methylophilales	Betaproteobacteria	1.38	71.86	0.049 *
SAR406 clade uncultured bacterium	Marinimicrobia	1.38	73.72	0.004 **
SAR406 clade	Marinimicrobia	1.38	75.58	0.004 **
SAR116 clade	Alphaproteobacteria	1.37	77.42	0.001 ***
Rickettsiales	Alphaproteobacteria	1.35	79.25	0.001 ***

Hydrogenophilaceae uncultured	Betaproteobacteria	1.32	81.03	0.001 ***
Rhizobiales	Alphaproteobacteria	1.32	82.80	0.002 **
Hydrogenophilaceae	Betaproteobacteria	1.30	84.55	0.001 ***
Hydrogenophilales	Betaproteobacteria	1.30	86.29	0.001 ***
Thiothrix	Gammaproteobacteria	1.24	87.96	0.010 **
Thiotrichaceae	Gammaproteobacteria	1.24	89.63	0.010 **

Pt15 - MIS

Biomarker		Contribution	Cumulative	P
Higher taxonomic rank	Phylum / Class	%	%	-
NS3a marine group	Flavobacteriia	2.30	56.78	0.001 ***

Pt15 - 6ext

Biomarker		Contribution	Cumulative	P
Higher taxonomic rank	Phylum / Class	%	%	-
NS3a marine group	Flavobacteriia	1.96	58.69	0.004 **
Formosa	Flavobacteriia	1.19	61.53	0.049 *

MIS - 6ext

Biomarker		Contribution	Cumulative	P
Higher taxonomic rank	Phylum / Class	%	%	-
Formosa	Flavobacteriia	1.26	59.85	0.046 *

Table S5. Results for variation partitioning with the environmental variables, which were selected by forward selection in the redundancy analyses (RDAs) (see Figure 4 in the main manuscript). The contribution (%) of each environmental variable and its significance are given.

Variable	Df	Variance	F	P
Residual	15	14,3275	-	-
**Mn**	1	1,2155	1,2726	**0.033 ***
**DOC**	1	1,2114	1,2682	**0.038 ***
**Salinity**	1	1,1169	1,1693	**0.032 ***
**Cu**	1	1,1144	1,1667	**0.047 ***
**Cd**	1	1,013	1,0606	**0.049 ***
Temperature	1	0,9304	0,9741	0.073
Cs	1	0,822	0,8605	0.087
Al	1	0,7796	0,8162	0.115
Fe	1	0,7228	0,7567	0.174
Li	1	0,6641	0,6953	0.135
Nanoeukaryotes	1	0,6113	0,64	0.243
Rb	1	0,6042	0,6326	0.234
V	1	0,563	0,5894	0.243
Chlorophyll *a*	1	0,554	0,58	0.325
Pb	1	0,5078	0,5316	0.338
Zn	1	0,4847	0,5075	0.369
TN	1	0,4753	0,4976	0.374
Ba	1	0,4714	0,4935	0.344
Sr	1	0,4324	0,4527	0.425
Picoeukaryotes	1	0,3852	0,4032	0.451

Table S6. Network analysis results, showing betweenness centrality (BC), closeness centrality (CC), number of connections (Degree) and relative abundance of keystone OTUs in the global network.

Kingdom	Class	Family	Genus	Label	BC	CC	Degree	Abundance
Bacteria	Alphaproteobacteria	Surface 1	uncultured bacterium	OTU-New.ReferenceOTU431	0.076	0.61	74	0.14
Bacteria	Alphaproteobacteria	Surface 1	uncultured bacterium	uncultured bacteriumcross233	0.046	0.57	64	0.14
Bacteria	Alphaproteobacteria	Surface 1	uncultured bacterium	OTU-New.ReferenceOTU241	0.068	0.55	68	0.20
Bacteria	Alphaproteobacteria	Surface 1	uncultured bacterium	uncultured bacteriumcross146	0.066	0.545	68	0.20
Bacteria	Actinobacteria	Microbacteriaceae	Candidatus Aquiluna	OTU-New.ReferenceOTU237	0.054	0.55	55	0.18
Bacteria	Cyanobacteria	FamilyI	Synechococcus	OTU-New.ReferenceOTU365	0.021	0.55	55	0.27
Bacteria	Bacteroidetes Incertae Sedis	Unknown Family	Balneola	uncultured bacteriumcross200	0.020	0.55	56	0.05
Bacteria	Actinobacteria1	Microbacteriaceae	Candidatus Aquiluna	uncultured bacteriumcross174	0.027	0.54	52	0.18
Bacteria	Cyanobacteria	FamilyI	Synechococcus	OTU-New.ReferenceOTU245	0.021	0.52	38	0.09
Bacteria	Alphaproteobacteria	Rhodobacteraceae	Planktomarina	OTU-New.ReferenceOTU394	0.024	0.51	46	0.0
Bacteria	Alphaproteobacteria	Rhodobacteraceae	uncultured	uncultured bacteriumcross104	0.048	0.51	35	0.21
Bacteria	Alphaproteobacteria	Rhodobacteraceae	Planktomarina	Planktomarina	0.027	0.51	46	0.30

Table S7. Fraction of OTUs not mapped to KEGG organisms (FTU) and percentage of OTUs mapped to KEGG organisms (%)

Sample	FTU	%
41pprof1	0,59	41,01
41pprof3	0,54	46,10
41pprof7	0,51	48,73
41pprof9	0,56	44,22
41pprof11	0,60	39,72
41pprof13	0,37	63,04
41pprof15	0,56	43,79
41pprof17	0,52	47,82
41pprof19	0,61	39,11
41pprof21	0,50	49,85
41pprof23	0,48	51,98
Pt15prof1	0,31	68,59
Pt15prof5	0,36	63,58
Pt15prof7	0,49	50,89
Pt15prof9	0,30	70,47
Pt15prof11	0,39	60,84
Pt15prof13	0,52	47,54
Pt12prof1	0,33	67,25
Pt12prof3	0,44	56,18
Pt12prof5	0,35	65,27
Pt12prof11	0,40	59,73
Pt12prof15	0,45	55,45
Pt12prof17	0,40	60,39
Pt12prof19	0,40	60,31
MISprof1	0,24	76,44
MISprof5	0,23	77,20
MISprof7	0,34	66,08
MISprof13	0,40	59,63
MISprof15	0,29	71,05
6extprof1	0,25	75,23
6extprof3	0,22	78,04
6extprof5	0,31	68,74
6extprof9	0,24	76,17
6extprof11	0,39	60,76
6extprof13	0,35	64,82
6extprof17	0,27	72,54
